# Longitudinal multiomic signatures of ARDS and sepsis inflammatory phenotypes identify pathways associated with mortality

**DOI:** 10.1172/JCI196290

**Published:** 2025-12-02

**Authors:** Narges Alipanah-Lechner, Lucile Neyton, Pratik Sinha, Carolyn Leroux, Kim Bardillon, Sidney A. Carrillo, Suzanna Chak, Olivia Chao, Taarini Hariharan, Carolyn Hendrickson, Kirsten Kangelaris, Charles R. Langelier, Deanna Lee, Chelsea Lin, Kathleen Liu, Liam Magee, Angelika Ringor, Aartik Sarma, Emma Schmiege, Natasha Spottiswoode, Kathryn Sullivan, Melanie F. Weingart, Andrew Willmore, Hanjing Zhuo, Angela J. Rogers, Kathleen A. Stringer, Michael A. Matthay, Carolyn S. Calfee

**Affiliations:** 1 Division of Pulmonary, Critical Care, Allergy, and Sleep Medicine, Department of Medicine, UCSF, San Francisco, California, USA.; 2 Division of Clinical and Translational Research, Department of Anesthesia, Washington University School of Medicine, St. Louis, Missouri, USA.; 3 Cardiovascular Research Institute, UCSF, San Francisco, California, USA.; 4 Division of Pulmonary and Critical Care, Department of Medicine, Zuckerberg San Francisco General Hospital, San Francisco, California, USA.; 5 Division of Hospital Medicine, Department of Medicine;; 6 Division of Infectious Disease, Department of Medicine;; 7 Division of Nephrology, Department of Medicine; and; 8 Department of Anesthesia, UCSF, San Francisco, California, USA.; 9 Division of Pulmonary and Critical Care, Department of Medicine, Stanford University, Stanford, California, USA.; 10 Department of Clinical Pharmacy, College of Pharmacy, and; 11 Division of Pulmonary and Critical Care Medicine and; 12 Weil Institute for Critical Care Research & Innovation, School of Medicine, University of Michigan, Ann Arbor, Michigan, USA.

**Keywords:** Clinical Research, Inflammation, Pulmonology, Metabolomics, Mitochondria, Transcriptomics

## Abstract

**BACKGROUND:**

Critically ill patients with acute respiratory distress syndrome (ARDS) and sepsis exhibit distinct inflammatory phenotypes with divergent clinical outcomes, but the underlying molecular mechanisms remain poorly understood. These phenotypes, derived from clinical data and protein biomarkers, were associated with metabolic differences in a pilot study.

**METHODS:**

We performed integrative multiomics analysis of blood samples from 160 patients with ARDS in the ROSE trial, randomly selecting 80 patients from each latent class analysis–defined inflammatory phenotype (hyperinflammatory and hypoinflammatory) with phenotype probability greater than 0.9. Untargeted plasma metabolomics and whole-blood transcriptomics at day 0 and day 2 were analyzed using multimodal factor analysis (MEFISTO). The primary outcome was 90-day mortality, with validation in an independent critically ill sepsis cohort (EARLI).

**RESULTS:**

Multiomics integration revealed 4 molecular signatures associated with mortality: (a) enhanced innate immune activation coupled with increased glycolysis (associated with hyperinflammatory phenotype), (b) hepatic dysfunction and immune dysfunction paired with impaired fatty acid β-oxidation (associated with hyperinflammatory phenotype), (c) interferon program suppression coupled with altered mitochondrial respiration (associated with hyperinflammatory phenotype), and (d) redox impairment and cell proliferation pathways (not associated with inflammatory phenotype). These signatures persisted through day 2 of trial enrollment. Within-phenotype analysis revealed distinct mortality-associated pathways in each group. All molecular signatures were validated in the independent EARLI cohort.

**CONCLUSION:**

Inflammatory phenotypes of ARDS reflect distinct underlying biological processes with both phenotype-specific and phenotype-independent pathways influencing patient outcomes, all characterized by mitochondrial dysfunction. These findings suggest potential therapeutic targets for precise treatment strategies in critical illness.

**FUNDING:**

NIH National Heart, Lung, and Blood Institute and National Institute of General Medical Sciences.

## Abstract 2

Multi-omics analysis of ARDS patients reveals four molecular signatures associated with death, all characterized by mitochondrial dysfunction and suggesting new therapeutic targets.

## Introduction

Acute respiratory distress syndrome (ARDS) and sepsis are devastating critical illness syndromes with unacceptably high mortality rates approaching 40%–50% in the United States (1, 2). A significant challenge to developing effective treatments has been the marked heterogeneity in clinical presentation, underlying biology, and treatment responses among affected patients (3, 4).

Recent advances in molecular phenotyping have identified reproducible subgroups of patients with ARDS and sepsis with distinct pathobiology. Latent class analyses (LCAs) of clinical and plasma protein data consistently reveal 2 predominant phenotypes: a “hyperinflammatory” phenotype characterized by elevated plasma inflammatory protein biomarkers, shock, and higher mortality and a “hypoinflammatory” phenotype with relatively lower inflammatory protein biomarkers and better outcomes (5–10). These phenotypes, identified across multiple ARDS and sepsis cohorts, demonstrate differential therapeutic responses in secondary analyses of randomized trials, suggesting they represent endotypes with distinct disease mechanisms (5, 11, 12). Clinical trials incorporating prospective phenotyping are being developed, including the PANTHER trial, which started enrollment in late 2025 (13). However, the biological processes driving each phenotype and mechanisms underlying unfavorable outcomes within each phenotype remain poorly understood. While protein biomarker studies have provided valuable insights into inflammatory patterns, they capture only a small fraction of the complex molecular landscape. Previous metabolic profiling of 93 patients with ARDS demonstrated that the hyperinflammatory phenotype exhibits reduced circulating lipids and a glycolytic shift, while transcriptomic analyses revealed increased expression of genes related to the innate immune response, tissue remodeling, and reduced interferon signaling (10, 14). However, isolated omic approaches may miss critical interactions between cellular programming and systemic metabolism essential for understanding disease processes and treatment responses.

In this study, we applied longitudinal multiomics profiling to characterize the molecular basis of ARDS/sepsis inflammatory phenotypes and identify mechanisms associated with poor outcomes. We hypothesized that these phenotypes would demonstrate distinct metabolic profiles and that integrated metabolomic-transcriptomic analysis would reveal novel outcome-associated mechanisms with therapeutic potential. By simultaneously measuring the metabolome and transcriptome at 2 time points in a large ARDS cohort, we aimed to (a) identify metabolic differences between inflammatory phenotypes, (b) characterize coordinated metabolomic-transcriptomic signatures contributing to heterogeneity, (c) determine temporal stability of these patterns, and (d) uncover potentially targetable pathways associated with mortality. This comprehensive molecular characterization aims to advance our understanding of ARDS/sepsis heterogeneity and identify therapeutic approaches tailored to specific patient subgroups.

## Results

### LCA phenotypes have distinct metabolic profiles.

**Figure 1:**
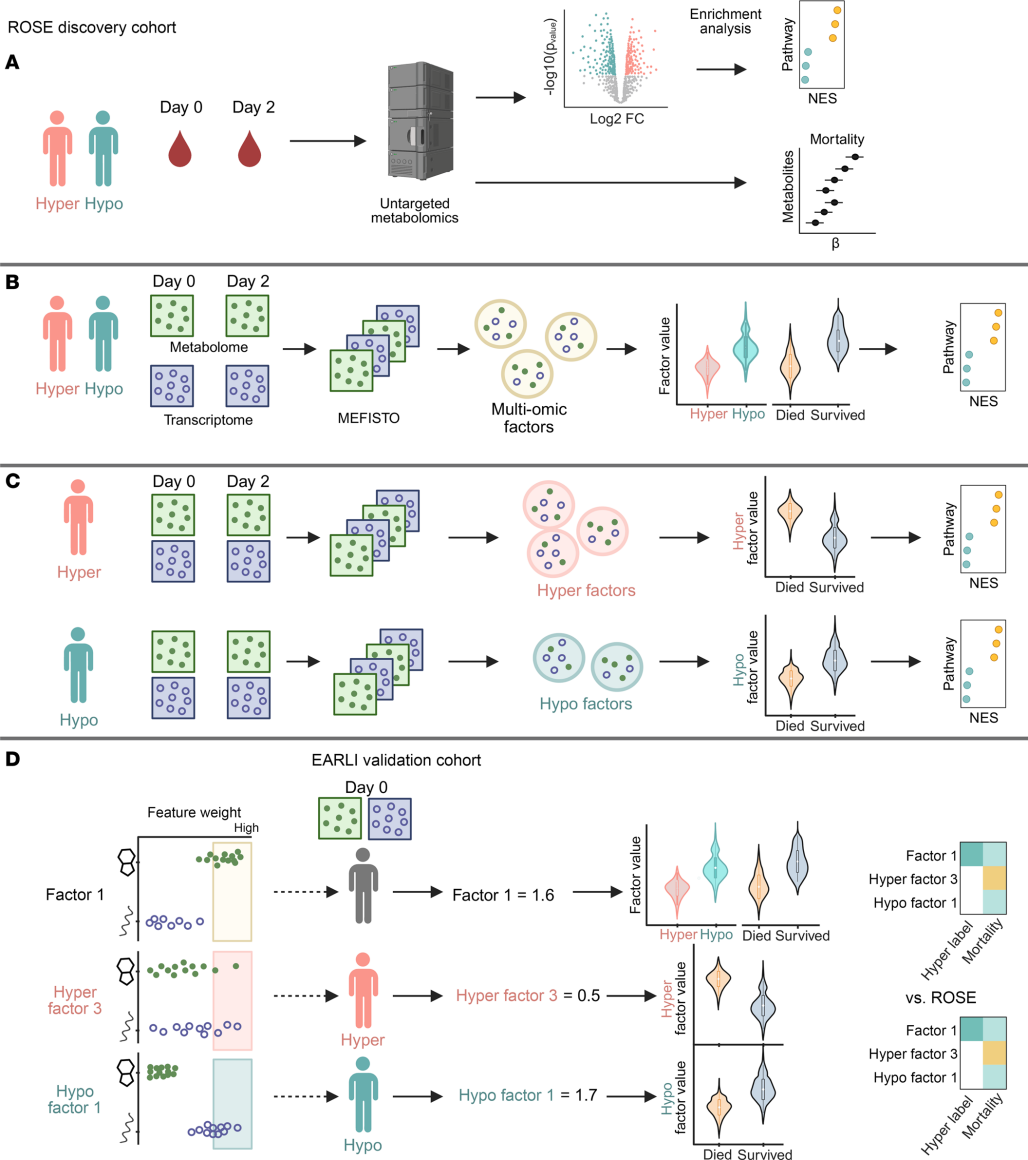
Study overview. (**A**) Day 0 and day 2 EDTA plasma from ROSE study participants underwent untargeted metabolic profiling to determine differences between latent class analysis (LCA) inflammatory phenotypes. (**B**) Longitudinal whole-blood transcriptomic data and metabolomic data were analyzed using an unsupervised multimodal factor analysis (MEFISTO), and the predominant sources of biological heterogeneity in the data of clinical relevance were assessed. (**C**) MEFISTO was applied separately to each phenotype to determine signatures related to mortality within each phenotype. (**D**) The highest weighted features (metabolite or gene) by absolute value within each multiomic factor of interest were used to calculate factor weights for patients in an observational cohort study (EARLI). The association of factor weights with LCA phenotypes and outcomes in the validation cohort was assessed. Created in BioRender. NES, normalized enrichment score.

We first asked whether ethylenediamine tetraacetic acid (EDTA) plasma metabolites would be different between LCA-defined ARDS phenotypes. We evaluated patients from the ROSE trial of neuromuscular blockade for the treatment of moderate-to-severe ARDS (Figure 1A), who had previously undergone LCA phenotyping using plasma protein biomarkers and clinical variables (10, 15). We randomly selected 80 patients in each phenotype with high phenotype membership probability (*P* > 0.9) (Supplemental Figure 1; supplemental material available online with this article; https://doi.org/10.1172/JCI196290DS1). These 160 patients had a median age of 58.5 (IQR 47 to 68), were predominantly male (64%), and were racially designated as White (78%), with equivalent proportions randomized to neuromuscular blockade across phenotypes (Supplemental Table 1). The hyperinflammatory group exhibited lower median body mass index (BMI), higher APACHE III scores, reduced glomerular filtration rate (GFR), and higher prevalence of comorbid liver disease and leukemia. Corticosteroid administration rates were identical between phenotypes (24%). Consistent with previous studies, hyperinflammatory patients more frequently required vasopressors at enrollment (86% vs. 21%), experienced more than twice the mortality at 28 and 90 days (61% vs. 24%), and had significantly fewer ventilator-, intensive care unit– (ICU-), and hospital-free days (Supplemental Table 1). Pneumonia was the predominant ARDS etiology in both phenotypes, while all patients with extrapulmonary sepsis-induced ARDS belonged to the hyperinflammatory group.

**Figure 2:**
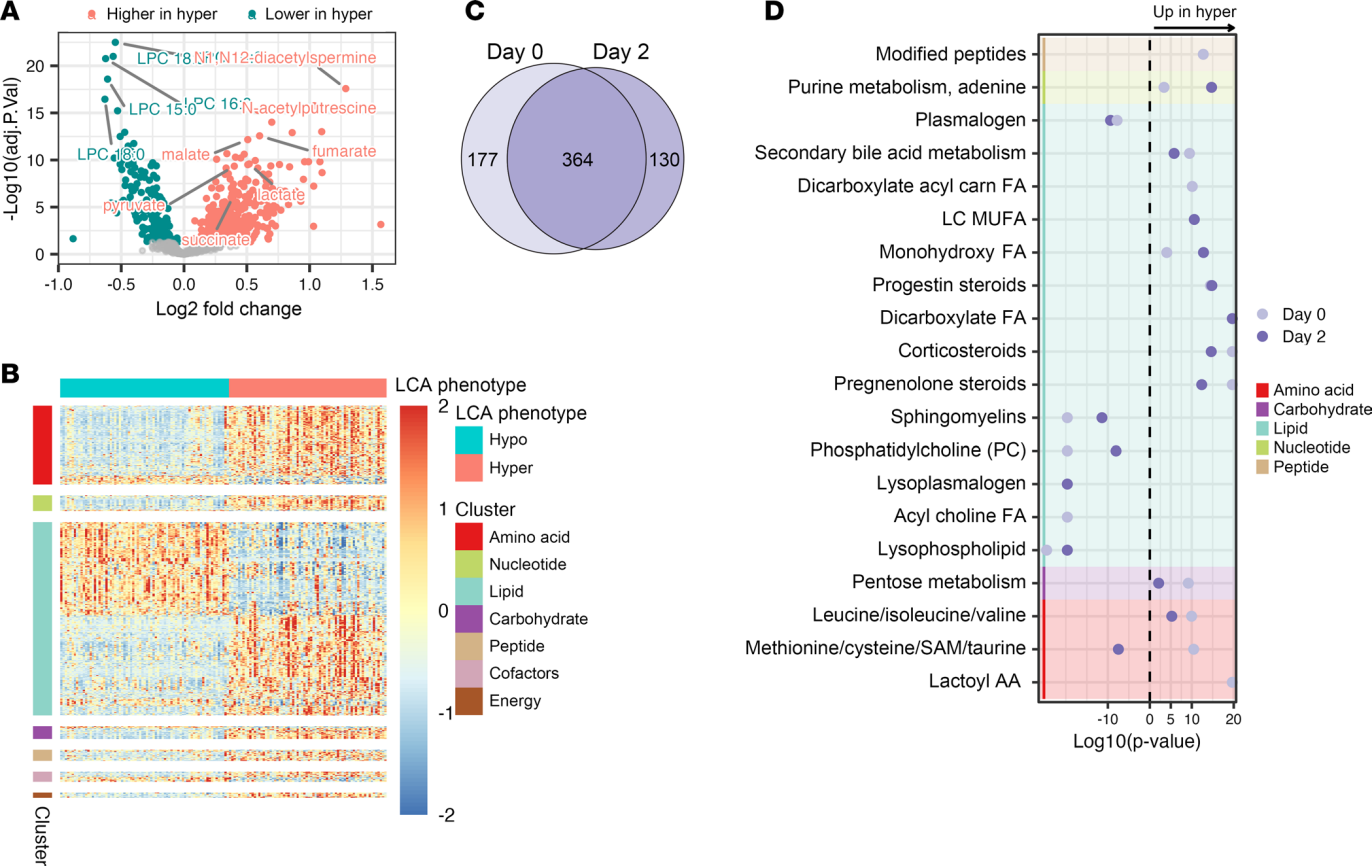
Metabolic profiling of LCA phenotypes (*N* = 160 patients). (**A**) Volcano plot showing differentially abundant metabolites between hyperinflammatory and hypoinflammatory ARDS at day 0, determined by limma adjusted for covariates (age, sex, BMI, medications, liver disease, and GFR). (**B**) Heatmap of differentially abundant metabolites by LCA phenotype at day 0 as determined by limma with adjustment for aforementioned covariates. *Z*-scaled log-transformed metabolite intensities are grouped by phenotype. (**C**) Venn diagram showing overlap of differentially abundant metabolites at day 0 and day 2 (day 2 also adjusted for randomization arm). (**D**) Metabolite pathway enrichment analysis comparing hyperinflammatory vs. hypoinflammatory groups at day 0 and day 2. *X* axis shows signed log_10_(*P* value), with positive values indicating positive enrichment in hyperinflammatory group and negative values indicating positive enrichment in hypoinflammatory group. Top 20 significant pathways are shown (FDR < 0.05). AA, amino acid; Carn, carnitine; FA, fatty acid; LC, long chain; MUFA, monounsaturated fatty acid; SAM, *S*-adenosylmethionine.

Untargeted metabolic profiling identified 1,378 known metabolites (Supplemental Figure 2). After removing metabolites with high missingness (>25%), 982 remained for analysis. Differential abundance analysis using limma with adjustment for potential confounders identified 541 metabolites significantly different between phenotypes at day 0, with substantial differences across all metabolic classes (Figure 2, A and B). Similar analysis at day 2 revealed 494 significantly different metabolites, largely overlapping with day 0 findings (Figure 2C). Metabolite enrichment analysis using Metabolon’s library highlighted 60 dysregulated pathways at day 0 and 56 at day 2, totaling 74 unique metabolic pathways (Figure 2D and Supplemental Figure 3). The top 20 most differentially abundant metabolites belonged to lipids and amino acid classes, though the highest proportion of differentially abundant metabolites were related to energy production at both time points (Supplemental Tables 2 and 3). The primary metabolic differences between phenotypes persisted in sensitivity analyses restricted to pneumonia-only patients and adjusting for shock (Supplemental Figure 4 and Supplemental Tables 4 and 5). Similarly, adjusting for renal replacement therapy did not meaningfully alter the metabolomic differences between phenotypes (Supplemental Figure 5 and Supplemental Tables 6 and 7).

In patients surviving through day 2, individual metabolite trajectories did not differ by 90-day mortality in the full cohort or within phenotypes (Supplemental Figure 6). However, when metabolites were aggregated by class, several metabolic classes demonstrated significantly different trajectories based on 90-day mortality (Supplemental Figure 7A). Tryptophan metabolism, steroid pathways, and γ-glutamyl amino acids increased over time in non-survivors, who also demonstrated decreasing levels of lactosylceramides, lysoplasmalogens, hexosylceramides, sphingolipids, phospholipids, and ascorbate/aldarate metabolites. Hypoinflammatory non-survivors had increasing progestin steroids (Supplemental Figure 7B), while hyperinflammatory non-survivors exhibited decreasing acyl carnitines, plasmalogens, and ascorbate/aldarate metabolites alongside rising pregnenolone steroids (Supplemental Figure 7C). Testing for sex interactions across all metabolites revealed no biologically meaningful sex-specific differences in mortality-related trajectories (Supplemental Figure 8).

### Mitochondrial metabolites are associated with hyperinflammatory phenotype and mortality.

**Figure 3:**
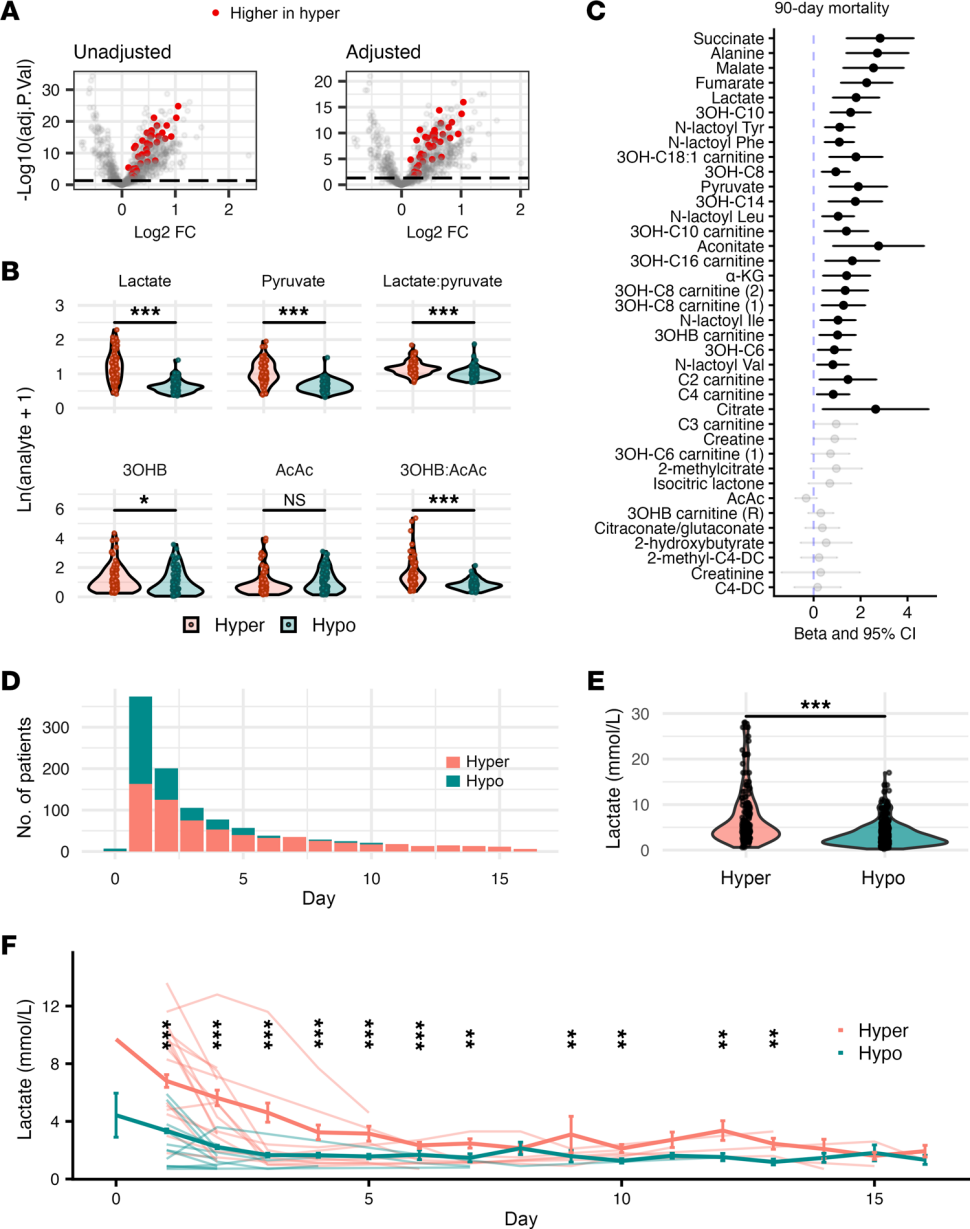
Mitochondrial metabolic derangements across LCA phenotypes. (**A**) Volcano plots comparing hyperinflammatory vs. hypoinflammatory phenotypes at day 0 in ROSE (*N* = 160 patients). Left: Unadjusted analysis showing statistical significance (limma) vs. log_2_ fold-change. Right: Analysis adjusted for age, sex, BMI, and vasopressor use. Solid colors represent mitochondrial metabolites. (**B**) Peak intensities of redox-coupled mitochondrial metabolites at day 0 in ROSE (*N* = 160 patients). (**C**) Association of mitochondrial metabolites at day 0 with 90-day mortality in ROSE patients (*N* = 160). *X* axis depicts regression coefficients with 95% confidence intervals from logistic regression models using log-transformed peak intensity as the primary predictor, adjusted for age, sex, BMI, and vasopressor use. Solid circles represent FDR *P* < 0.05. (**D**) Distribution of patients with clinical lactate measurements by LCA phenotype and enrollment day in the EARLI cohort (*N* = 546, hypoinflammatory: 380, hyperinflammatory: 166). (**E**) Comparison of highest clinical lactate value (days 0–2 after enrollment) per patient by phenotype in EARLI. (**F**) Longitudinal clinical lactate trajectories by phenotype showing mean ± SEM. Individual patient trajectories (random sample) are depicted, excluding those with lactate value > 15 mmol/L for visualization clarity. Wilcoxon’s rank-sum test *P* < 0.05 shown for each time point. *P < 0.05, **P < 0.01, ***P < 0.001. 3OHB, 3-hydroxybutyrate; FC, fold-change.

We hypothesized that observed derangements in fatty acid oxidation, lactoyl amino acids, and TCA metabolites stemmed from mitochondrial dysfunction. To test this hypothesis, we curated mitochondria-associated metabolites based on established circulating biomarkers in genetic mitochondrial disorders (16). Of 38 detectable mitochondria-associated metabolites in our cohort, 37 (97%) differed significantly between phenotypes (Figure 3A). Since vasopressors can enhance glycolysis and lactate production (17, 18), we investigated whether increased mitochondrial metabolic activity in the hyperinflammatory group merely reflected vasopressor administration. Differential abundance analysis incorporating vasopressor administration (>1-hour infusion in preceding 24 hours) as a covariate revealed that 31 (81%) mitochondrial metabolites remained differentially abundant between phenotypes (Figure 3A), suggesting the distinct mitochondrial signature in the hyperinflammatory phenotype is independent of vasopressor effects. Further examining metabolic mitochondrial function through plasma redox-coupled (e.g., NADH/NAD^+^) metabolite pairs (19–23), we observed both lactate/pyruvate and 3-hydroxybutyrate/acetoacetate ratios were significantly higher in hyperinflammatory patients (Figure 3B), indicating systemic redox imbalance. Finally, assessing clinical relevance, 26 of 38 mitochondrial metabolites (68%) were associated with 90-day mortality in multivariate logistic regression models (Figure 3C).

As proof of concept that metabolic differences identified through untargeted profiling reflected clinically quantifiable phenotype distinctions, we tested whether clinical lactate values differed by phenotype. Since ROSE lacked clinical lactate data, we examined measurements from EARLI, an independent cohort of critically ill patients with sepsis who had undergone LCA phenotyping (24). While both phenotypes presented with elevated lactate levels, hyperinflammatory patients had persistently higher lactate throughout nearly the entire follow-up period (Figure 3, D–F), validating that our metabolomic approach successfully identified clinically meaningful phenotypic differences. To further validate that metabolomic measurements captured patient-level lactate differences, we compared rankings between metabolomic and clinical lactate within EARLI patients with paired measurements available at baseline (*n* = 137). Metabolomic lactate demonstrated strong rank correlation with clinical lactate (Pearson’s p = 0.615; *P* < 1 × 10^–4^; Supplemental Figure 9), confirming our untargeted platform reliably captures relative metabolite differences between patients.

### Multiomics analysis identifies principal factors related to LCA phenotypes.

**Figure 4:**
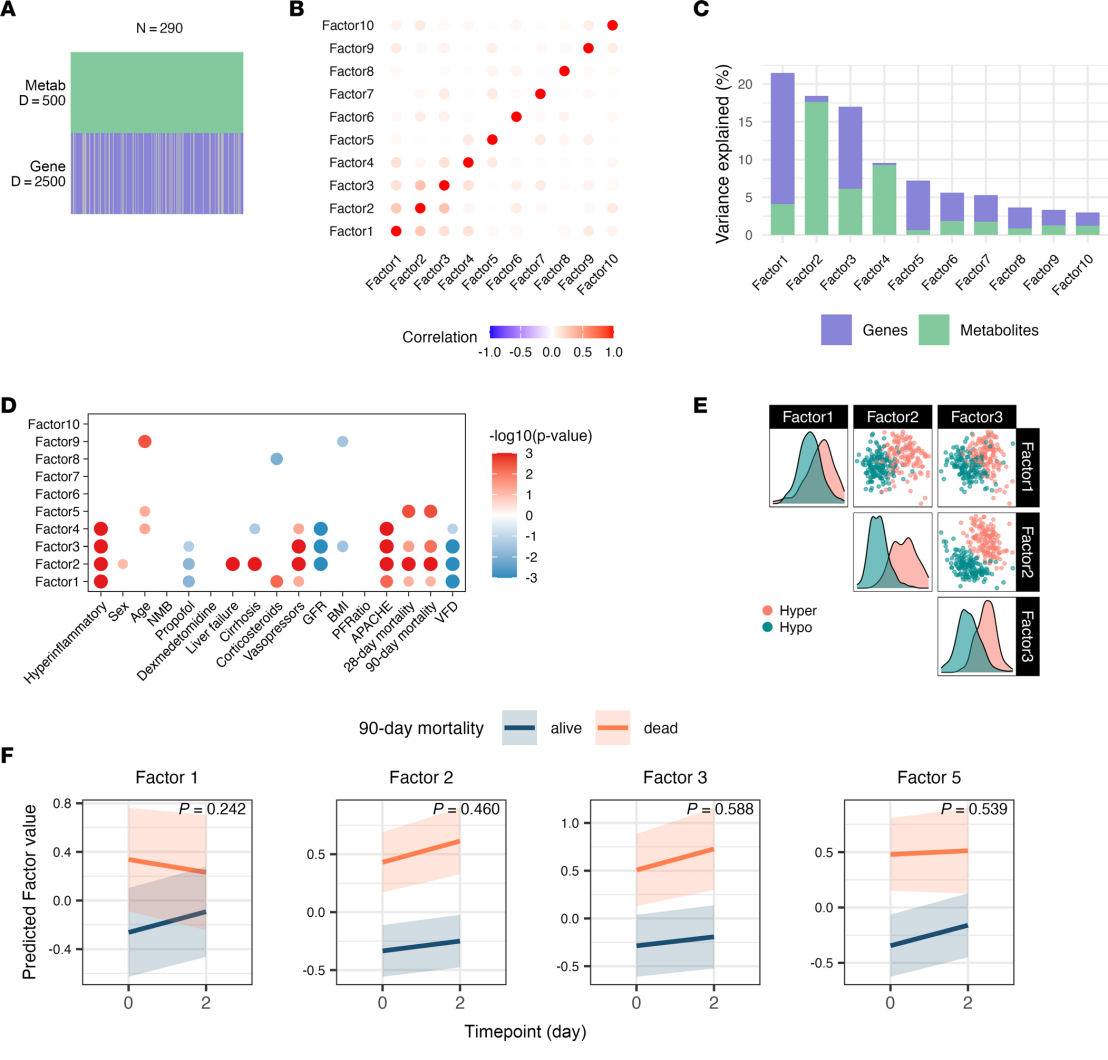
MEFISTO overview and association with clinical outcomes. (**A**) MEFISTO model overview with 290 samples (from 160 patients across 2 time points), top 500 metabolites, and top 2,500 gene transcripts by median absolute deviation. (**B**) Spearman’s correlation plot of 10 MEFISTO factors based on factor values. (**C**) Proportion of total variance explained by each factor and each data modality (metabolite vs. gene transcript). (**D**) The association of MEFISTO factor values at day 0 with clinical variables. Size and transparency of the dots represent strength of association as determined via Spearman’s correlation for continuous predictors or linear regression for categorical predictors (FDR < 0.05). Color represents directionality of the correlation. (**E**) Paired plots of MEFISTO factor values per patient sample, colored by LCA phenotype designation. (**F**) The slope of change in factor values over time by survival. Only factors associated with mortality at day 0 are depicted. *P* value derived from interaction term of a linear mixed effects regression model with 90-day mortality, time point, and their interaction as fixed effects and patient as random effect. APACHE, Acute Physiology and Chronic Health Evaluation III score; BMI, body mass index; GFR, glomerular filtration rate; metab, metabolite; NMB, neuromuscular blockade; PFRatio, PaO_2_/FiO_2_ at the time of enrollment in the ROSE trial; VFD, ventilator-free days.

To identify principal sources of biological heterogeneity in the ROSE cohort, we next performed integrated analysis of longitudinal metabolomics and whole-blood transcriptomics across all patients (Figure 1B). We selected the top 500 metabolites and 2,500 gene transcripts by median absolute deviation in the full cohort for multiomics analysis (Figure 4A). Applying a MEFISTO (Method for the Functional Integration of Spatial and Temporal Omics data) model incorporating both data types from both time points, we used temporal information as a covariate and configured the model to identify 10 latent factors (25). MEFISTO is a dimensionality reduction unsupervised approach for integrating multimodal data to identify driving sources of variation across data modalities. MEFISTO also disentangles sources of variation that change over time from those that are independent of time. Though MEFISTO does not enforce factor orthogonality, Spearman’s correlation analysis revealed no significant interfactor correlations, confirming each factor captured a distinct source of variability (Figure 4B). The model explained 49.6% of the total variance (*R*^2^) in transcriptomic data and 40.6% in metabolomic data (Supplemental Figure 10). Factors 1–3 collectively accounted for 59% of explained transcriptomic variance and 69% of explained metabolomic variance (Figure 4C and Supplemental Table 8). Factor 1 was predominantly driven by transcriptomic data, factor 2 by metabolomic data, and factor 3 by both data modalities.

We next analyzed associations between each latent factor at day 0 and key clinical characteristics and outcomes. Factors 1–4 exhibited strong associations with LCA phenotype designation, APACHE III scores, and ventilator-free days, while demonstrating variable associations with GFR, vasopressor use, corticosteroid administration, and propofol infusion (Figure 4D). The first 3 factors each explained more than 15% of model variance and were independently associated with mortality. Notably, factor 5, lacking association with LCA phenotypes, demonstrated strong independent association with mortality. While factor 2 substantially separated phenotypes, the combination of factors 2 and 3 achieved near-complete phenotype discrimination (Figure 4E). These findings indicate that the principal sources of biological heterogeneity identified through our data-driven multiomic approach strongly aligned with the biological signals captured by LCA phenotype designation.

### Multiomic factors are related to mortality.

**Figure 5:**
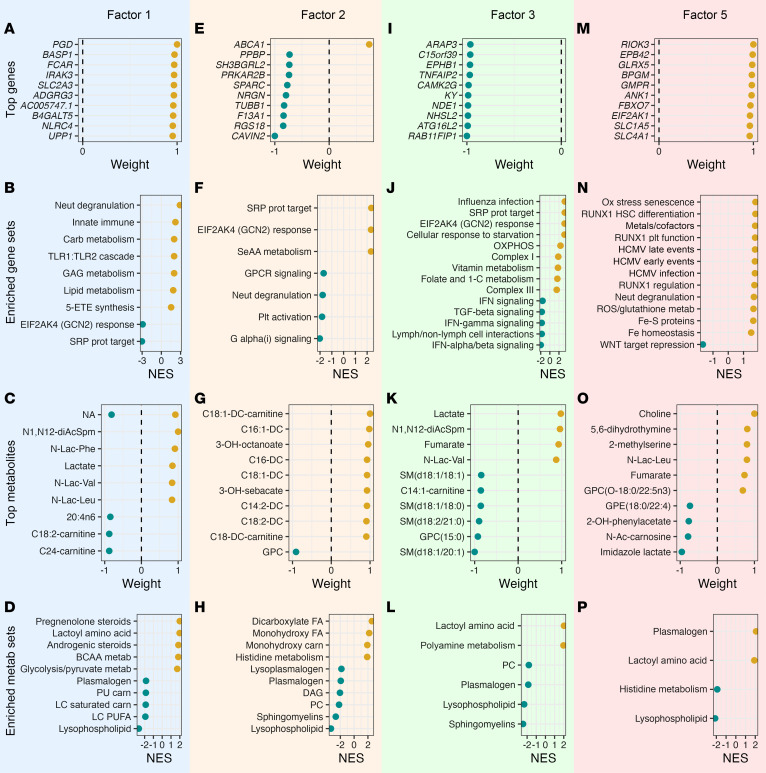
Top features and pathways within mortality-associated MEFISTO factors. (**A**, **E**, **I**, and **M**) Top 10 genes by relative scaled weight in each factor. (**B**, **F**, **J**, and **N**) Select top gene set enrichment pathways in each factor using Reactome pathways in each factor using Reactome and MitoCarta 3.0. *X* axis depicts normalized enrichment score (NES). (**C**, **G**, **K**, and **O**) Top 10 metabolites by relative scaled weight in each factor. (**D**, **H**, **L**, and **P**) Top metabolic pathways. *X* axis depicts NES. 5-ETE, 5-eicosatetraenoic acids; BCAA, branched chain amino acid; carb, carbohydrate; carn, carnitine; DAG, diacylglycerols; FA, fatty acid; GAG, glycosaminoglycan; GPCR, G protein–coupled receptor; HSC, hematopoietic stem cell; IFN, interferon; LC, long chain; Metab, metabolism; Neut, neutrophil; plt, platelet; Ox, oxidative; OXPHOS, oxidative phosphorylation; PUFA, polyunsaturated fatty acid; SeAA, selenoamino acid; ROS, reactive oxygen species; SRP, signal recognition particle; TLR, Toll-like receptor.

Factors 1, 2, 3, and 5 demonstrated strong associations with mortality (Figure 4D). MEFISTO identified all factors as time independent (time scales = 0), and the rate of change in factor values over time did not differ by 90-day mortality outcome (Figure 4F). In stepwise logistic regression analysis, a combination of factors 2 and 3 was sufficient to nullify the relationship between LCA phenotype and mortality (Supplemental Figure 11). Complete enrichment results are provided in the Supporting Data Values, with representative examples shown in Figure 5.

Factor 1, predominantly driven by gene expression, revealed coordinated changes between whole-blood transcripts and plasma metabolites, primarily reflecting innate immune activation (Figure 5, A–D). To better understand the cellular origins of these transcriptional signatures, we performed computational deconvolution using CIBERSORTx with a published sepsis neutrophil reference dataset (26). Factor 1 demonstrated strong positive correlation with total neutrophils and immature progenitor neutrophils, while showing negative correlation with adaptive immune cells (Supplemental Figure 12A). Gene set enrichment analysis demonstrated significant positive enrichment in neutrophil degranulation, characterized by upregulation of emergency granulopoiesis markers and stress response genes (Supplemental Figure 12, B–D), alongside TLR1/TLR2 signaling pathways, GAG metabolism, lipid metabolism, and 5-ETE synthesis pathways, with negative enrichment in protein synthesis/trafficking and EIF2AK4-mediated integrated stress response pathways. These transcriptional changes accompanied systemic metabolic alterations characterized by decreased plasma levels of long-chain polyunsaturated fatty acids, lysophospholipids, and plasmalogens, coupled with elevated pregnenolone and androgenic steroids, lactoyl amino acids, glycolytic intermediates, and branched chain amino acid catabolites.

Factor 2, significantly associated with clinical evidence of hepatic and renal dysfunction (Figure 4D), was primarily metabolite driven (Figure 5, E–H). The plasma metabolome demonstrated accumulation of ω-oxidation products (monohydroxy and dicarboxylated fatty acids) alongside decreased membrane-associated lipids and lipid signaling molecules (Figure 5H). Transcriptional profiling revealed increased expression of ABCA1, the cholesterol efflux pump, as well as positive enrichment of translation machinery and EIF2AK4-mediated amino acid stress response pathways (Figure 5F), with negative enrichment in neutrophil degranulation (Supplemental Figure 12), platelet activation, and GPCR signaling pathways. Computational deconvolution revealed that factor 2 correlated negatively with mature neutrophils and adaptive immune cells, suggesting depletion or functional suppression of these populations (Supplemental Figure 12).

Factor 3, associated with clinical evidence of renal dysfunction (Figure 4D), was characterized by impaired host response with reduced interferon signaling and increased systemic metabolic stress (Figure 5, I–L). Transcriptional analysis revealed positive enrichment for influenza infection and basic cellular processes, including protein synthesis and RNA processing, while immune signaling pathways were broadly suppressed (Figure 5J). Higher factor 3 values corresponded with increased expression of mitochondrial oxidative phosphorylation genes, particularly complexes I and III. Notably, both type I and type II interferon signaling pathways were downregulated, alongside decreased expression of lymphoid cell interaction genes. These transcriptional changes were accompanied by elevated lactoyl amino acids and polyamines and reduced sphingomyelins and lysophospholipids.

Factor 5 values were significantly associated with mortality but not LCA phenotype (Figure 4D). Analysis revealed a molecular state characterized by cell proliferation and oxidative stress (Figure 5, M–P). Transcriptional profiling demonstrated positive enrichment of DNA replication, cell cycle progression, RUNX1-mediated hematopoietic differentiation and megakaryocyte activation, HCMV infection, and increased WNT target gene engagement (Figure 5N). This hyperproliferative state featured increased expression of mitochondrial iron homeostasis genes, Fe-S protein metabolism, and ROS management systems, concurrent with activation of oxidative stress–induced senescence pathways.

### Multiomics analysis reveals mortality-associated signatures within LCA phenotypes.

**Figure 6:**
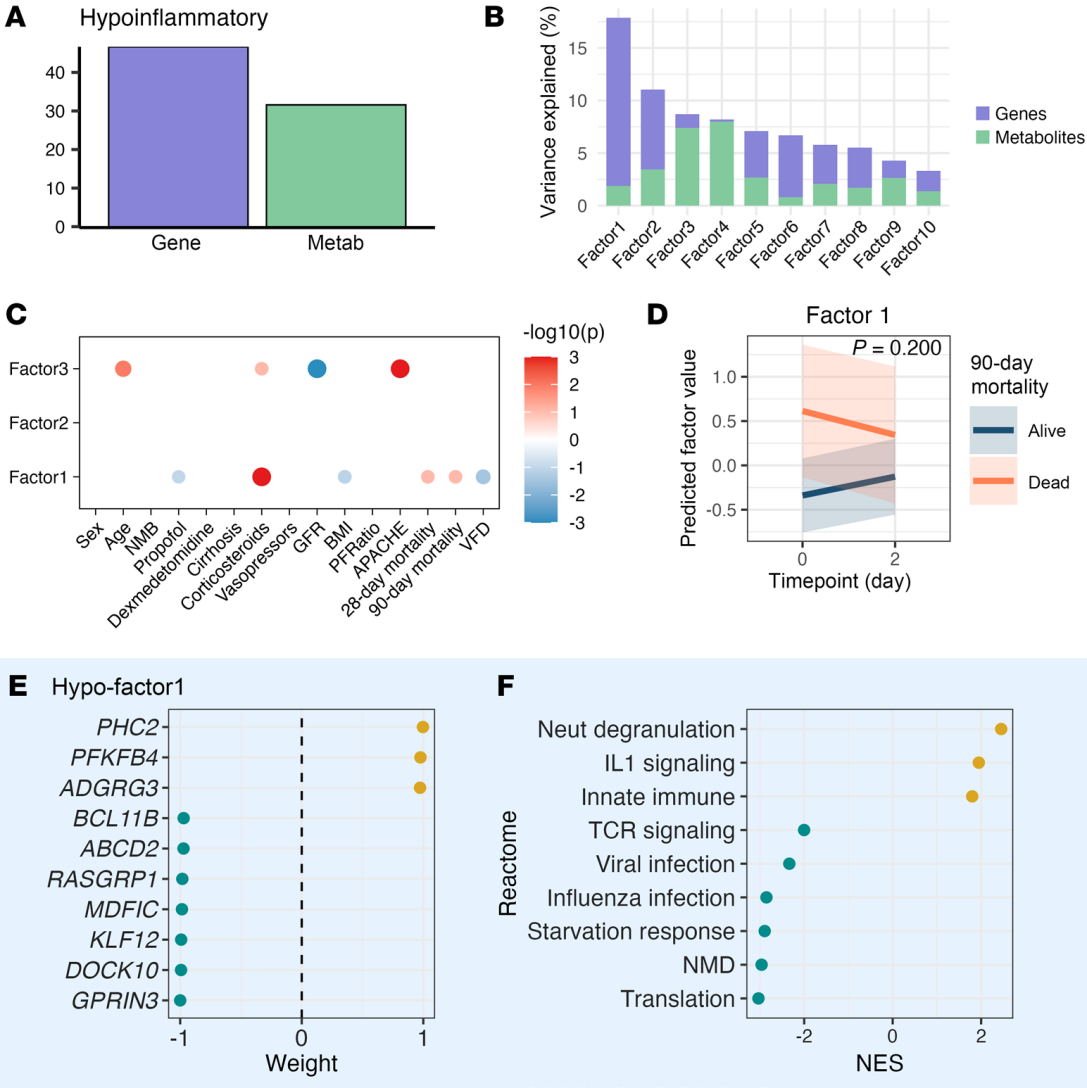
Hypoinflammatory MEFISTO and mortality-associated signature. Analysis of 153 samples from 80 patients at 2 time points. (**A**) Proportion of total variance explained per MEFISTO factor and per data modality (gene, metabolite). (**B**) Proportion of total variance explained per MEFISTO factor. (**C**) Association of MEFISTO factors with clinical variables at day 0 as determined via Spearman’s correlation for continuous predictors and linear regression for categorical predictors (FDR < 0.05). (**D**) The slope of change in factor 1 over time by 90-day mortality. *P* value derived from interaction term of a linear mixed effects regression model with 90-day mortality, time point, and their interaction as fixed effects and patient as random effect. (**E**) Top genes in factor 1 by relative scaled weight. (**F**) Enriched gene expression pathways in factor 1. *X* axis depicts normalized enrichment score (NES). NMD, nonsense-mediated decay; APACHE, Acute Physiology and Chronic Health Evaluation III score; BMI, body mass index; GFR, glomerular filtration rate; IL, interleukin; metab, metabolite; Neut, neutrophil; NMB, neuromuscular blockade; NMD, nonsense-mediated decay; PFRatio, PaO_2_/FiO_2_ at the time of enrollment; TCR, T cell receptor; VFD, ventilator-free days.

**Figure 7:**
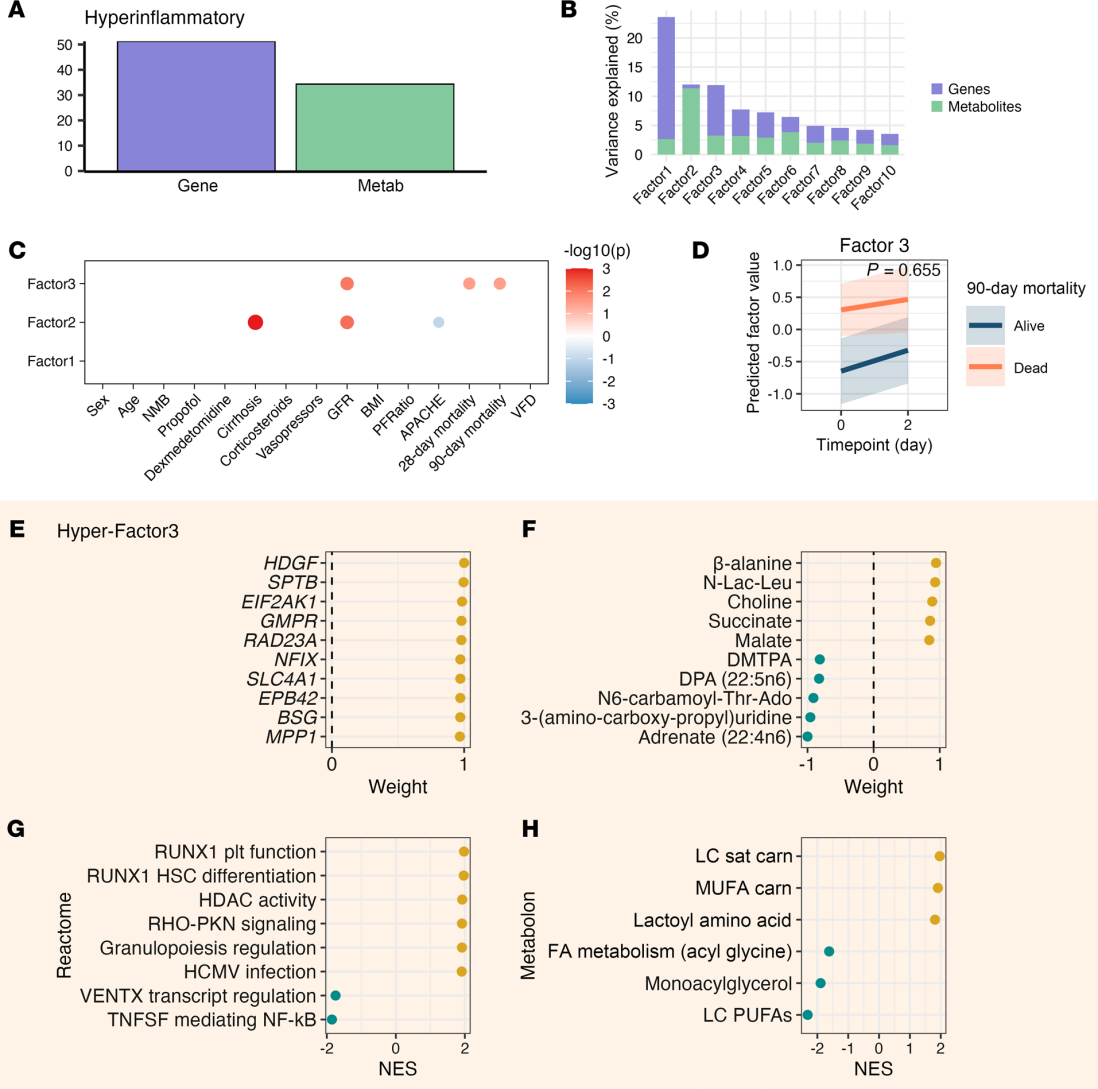
Hyperinflammatory MEFISTO and mortality-associated signature. Analysis of 137 samples from 80 patients at 2 time points. (**A**) Proportion of total variance explained per data modality (gene, metabolite). (**B**) Proportion of total variance explained per MEFISTO factor. (**C**) Association of MEFISTO factors with clinical variables at day 0 as determined via Spearman’s correlation for continuous predictors and linear regression for categorical predictors (FDR < 0.05). (**D**) The slope of change in factor 3 over time by 90-day mortality. *P* value derived from interaction term of a linear mixed effects regression model with 90-day mortality, time point, and their interaction as fixed effects and patient as random effect. (**E**) Top genes in factor 3 by relative scaled weight. (**F**) Top metabolites in factor 3 by relative scaled weight. (**G**) Top enriched gene expression pathways by normalized enrichment score (NES) in factor 3. (**H**) Top enriched metabolic pathways by NES in factor 3. ESR, estrogen receptor; plt, platelet; APACHE, Acute Physiology and Chronic Health Evaluation III score; BMI, body mass index; Carn, carnitine; FA, fatty acid; GFR, glomerular filtration rate; HSC, hematopoietic stem cell; LC, long chain; metab, metabolism; MUFA, monounsaturated fatty acid; PUFA, polyunsaturated fatty acid; Sat, saturated.

To investigate mechanisms underlying outcome heterogeneity within each ARDS phenotype, we conducted separate multiomics factor analyses within each phenotype (Figure 1C). Using MEFISTO with identical parameters to our full cohort analysis, we found that, in both phenotypes, transcriptional variation contributed more substantially to within-phenotype heterogeneity than metabolomic variation (Figure 6, A and B, and Figure 7, A and B).

In the hypoinflammatory group, factor 1, primarily characterized by gene expression patterns, was associated with mortality (Figure 6, B and C). Factor 1 values demonstrated no differential change over time based on survival status (Figure 6D) but had strong association with moderate-to-high dose of corticosteroid treatment in the preceding 24 hours. Gene expression profiling revealed positive enrichment of innate immune response pathways (neutrophil degranulation and IL-1 signaling) with concurrent negative enrichment of translation machinery, starvation response, nonsense-mediated decay, viral infection, adaptive immune response, and integrated stress response pathways among others (Figure 6, E and F).

In the hyperinflammatory group, factors 1–3 explained most data variance (Figure 7B). While factor 1 had no association with clinical variables, factor 3 demonstrated strong association with mortality, with similar temporal trajectories between survivors and non-survivors (Figure 7, C and D). Factor 3 was characterized by elevated expression of genes involved in RUNX1-mediated hematopoiesis and megakaryopoiesis, epigenetic remodeling, viral infection signatures, and increased cell cycle activity with negative enrichment in transcriptional regulation by VENTX and TNF receptor superfamily mediating non-canonical NF-κB pathways (Figure 7, E and G). TCA cycle intermediates and mitochondrial metabolites (malate, succinate, fumarate, lactate) were positively weighted (Figure 7F). Metabolite analysis identified systemic stress markers (lactoyl amino acids), altered lipid metabolism, and reduced long-chain polyunsaturated fatty acids among others (Figure 7H).

### Multiomic signatures are validated in external cohorts.

To assess generalizability of MEFISTO latent factors derived from our cohort with extreme phenotype designations, we examined these associations in EARLI, an ongoing, prospective, observational cohort study of critically ill adults with sepsis (Figure 1D). A subset of EARLI patients meeting sepsis criteria within 2 days of enrollment (*n* = 818) had previously undergone LCA phenotyping (24). Metabolomic data were available for 195 patients, whole-blood transcriptomics for 196 patients, and both data types for 61 patients (Supplemental Figure 13 and Supplemental Table 9) (27, 28).

**Figure 8:**
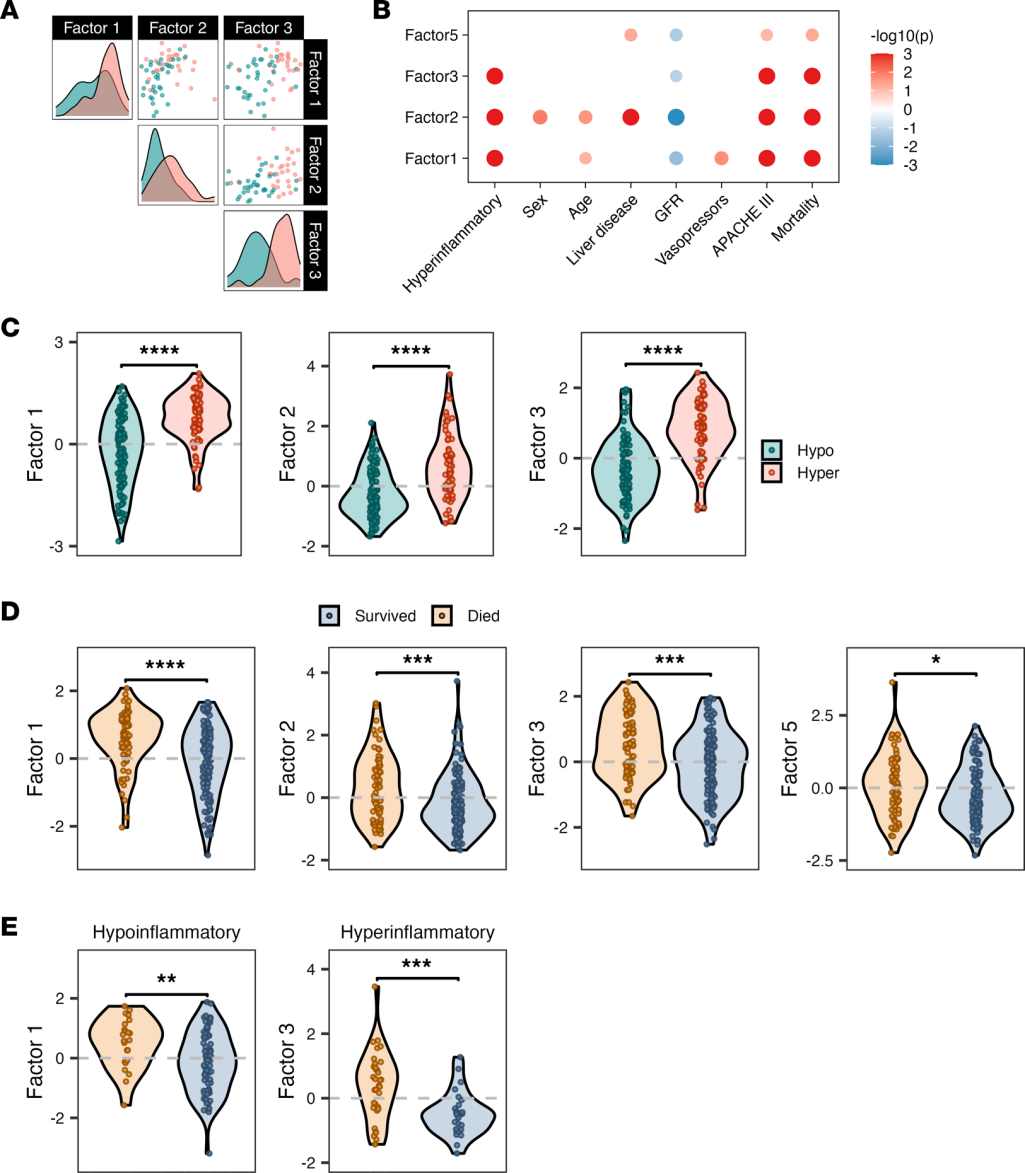
ROSE MEFISTO factor projections in the EARLI observational cohort. (**A**) Scatterplot of paired projected factor values per patient sample, colored by LCA phenotype designation, in patients with both transcriptomic and metabolomic data available (*N* = 61). (**B**) Association of projected MEFISTO factor values with clinical variables as determined via Spearman’s correlation for continuous predictors and linear regression for categorical predictors. (**C**) Projected factor values per patient sample comparing hyperinflammatory and hypoinflammatory phenotype. *P* value determined by Wilcoxon’s rank-sum. *N* = 189 patients with transcriptomic data (factors 1 and 3), and *N* = 183 patients with metabolomic data (factor 2). (**D**) Projected factor values per patient sample comparing hospital survivors and non-survivors in those with physician-adjudicated sepsis and transcriptomic data (*N* = 196 for transcriptomic factors 1, 3, and 5; *N* = 195 for metabolomic factor 2). *P* value determined by Wilcoxon’s rank-sum. (**E**) Projected phenotype-specific MEFISTO factors associated with mortality onto the extreme (*P* > 0.9) hypoinflammatory (left, *N* = 101) and extreme hyperinflammatory (right, *N* = 61) patients in EARLI. *P < 0.05, **P < 0.01, ***P < 0.001, ****P < 0.0001. APACHE, Acute Physiology and Chronic Health Evaluation III score; GFR, glomerular filtration rate.

To project ROSE MEFISTO factors onto EARLI patients, we selected the top 100 highest weighted features by absolute scaled weight within each factor of interest, yielding 2 transcriptomic signatures (factors 1 and 3) and 1 metabolomic signature (factor 2). This approach reduced noise from lower-weighted features and enabled testing in a larger cohort. We calculated factor 1 and 3 scores for EARLI patients with transcriptomic data (*n* = 196) and factor 2 values for those with metabolomic data (*n* = 195). All 3 ROSE MEFISTO factors demonstrated similar LCA phenotype associations in EARLI, with improved phenotype discrimination achieved by combining factors 2 and 3 (Figure 8, A–C). The 4 mortality-associated ROSE MEFISTO factors were similarly associated with mortality in EARLI (Figure 8D).

Using the same approach, we projected ROSE MEFISTO mortality-associated factors derived within each LCA phenotype onto the EARLI participants with high phenotype probability (*P* > 0.9). The top 100 features in each phenotype-specific factor yielded 1 transcriptomic signature per phenotype. Among patients with phenotype probability greater than 0.9, transcriptomic data were available for 101 with hypoinflammatory and 61 with hyperinflammatory sepsis. Both phenotype-specific mortality signatures demonstrated significant mortality associations in the EARLI cohort (Figure 8E).

## Discussion

In this integrated multiomic analysis of ARDS inflammatory phenotypes, we identified distinct transcriptional and metabolomic signatures that differentiate hyperinflammatory from hypoinflammatory phenotypes and are associated with clinical outcomes. Three key insights emerged: First, the hyperinflammatory phenotype exhibits profound mitochondrial dysfunction and metabolic derangement associated with mortality, persisting independently of vasopressor use, suggesting an intrinsic phenotypic feature. Second, longitudinal multiomic integration revealed 4 mortality-associated molecular factors representing distinct pathobiological processes: (a) innate immune activation with enhanced glycolysis, (b) hepatic dysfunction coupled with impaired fatty acid β-oxidation, (c) suppressed interferon signaling with altered mitochondrial respiration, and (d) immune cell proliferation with redox stress. Third, we identified biological signals associated with mortality within each inflammatory phenotype and quantified their relative contribution to overall biological heterogeneity and temporal evolution. These molecular signatures were replicated in an independent cohort of critically ill patients with sepsis, indicating their generalizability. Together, these findings advance our understanding of ARDS and sepsis heterogeneity and identify potential therapeutic targets for phenotype-specific interventions.

Factor 1, accounting for the largest proportion of molecular variation (35% transcriptomic, 10% metabolomic variance), reveals crucial insights into the relationship between inflammation and outcomes in ARDS. This factor represents an enhanced innate immune response through neutrophil activation and TLR1/TLR2 signaling, coupled with hypermetabolism. The increased expression of genes related to synthesis of inflammatory mediators (5-ETE) combined with reduced plasma PUFA levels suggest active consumption of circulating lipids, likely to support increased energy demands of expanding immune cell populations and generation of lipid mediators. GAG metabolism enrichment suggests tissue remodeling and altered barrier function, while elevated lactoyl amino acids and increased glycolysis suggest widespread mitochondrial metabolic stress and potential Warburg effect, or aerobic glycolysis, as this factor was independent of hypoxia status (PaO_2_/FiO_2_) (16, 29). Together, these findings suggest a coordinated systemic response where circulating immune cells undergo inflammatory expansion with corresponding metabolic adaptation via increased glycolysis and lipid metabolism. While our observational data cannot establish whether metabolic disturbances drive immune activation or vice versa, existing literature indicates these relationships are likely bidirectional. Metabolic conditions can modulate immune cell gene expression through epigenetic modifications and transcription factor activation, while immune cell activation drives metabolic reprogramming through altered enzyme expression and activity (30–34). For instance, neutrophil activation involves glycolytic reprogramming to support effector functions, while metabolites like lactate and succinate can directly influence immune cell gene transcription and inflammatory responses through HIF-1α and other metabolic sensors (31, 35–37). Notably, factor 1 had the weakest association with mortality, suggesting that interventions solely targeting broad suppression of inflammatory responses may be insufficient to fundamentally reduce mortality related to ARDS and sepsis. Indeed, the stronger signatures of mortality in this cohort were related to factors 2 and 3, both characterized by attenuated immune responses.

A consistent mortality signal in our analyses was related to renal and hepatic dysfunction coupled with impaired fatty acid β-oxidation (factor 2), strongly associated with the hyperinflammatory phenotype. Dicarboxylic fatty acids (DCFAs) are generated primarily in liver and kidney through ω-oxidation, an alternative pathway that metabolizes excess fatty acids when mitochondrial β-oxidation is compromised (38, 39). Elevated DCFAs, typically detected in urine of patients with mitochondrial fatty acid oxidation disorders, can further impair mitochondrial respiration and ATP synthesis via mitochondrial uncoupling (40, 41). The combination of elevated DCFAs and low plasmalogen levels also suggests peroxisomal dysfunction, as DCFAs undergo preferential peroxisomal β-oxidation, and peroxisomes are essential for plasmalogen biosynthesis (42, 43). Peroxisomes also play a crucial role in regulating inflammation by maintaining neutrophil membrane phospholipid composition and viability. Together, this metabolic signature, with its persistent elevation over time in non-survivors, implies liver and kidney dysfunction leading to metabolic derangements that could further exacerbate end organ dysfunction and contribute to impaired immunity, creating a vicious cycle strongly associated with mortality. Therapeutic interventions targeting lipid homeostasis restoration, such as l-carnitine supplementation, plasmalogen replacement, or simvastatin, could be candidates for study in this patient population (43–45).

Factor 3, strongly associated with both the hyperinflammatory phenotype and mortality, represents broad impairment in host response with reduced interferon signaling (type I and type II) and lymphoid cell interactions, alongside enrichment of integrated stress response pathways, influenza infection, increased cell turnover, and altered mitochondrial respiration. Suppressed type I interferon responses have been documented in peripheral blood of patients with severe COVID-19, in monocytes from bronchoalveolar lavage of patients with COVID-19/metapneumovirus coinfection, and in pediatric patients with severe respiratory syncytial virus infection (46–48). Similarly, reduced interferon signaling was observed in the MARS1 transcriptional phenotype of critically ill patients with sepsis at highest mortality risk (49). Whether this broad interferon program suppression results from pathogen-specific mechanisms or host biological heterogeneity remains unclear. Therapeutic interferon-γ has shown promise in sepsis-induced immunosuppression, particularly benefiting patients with decreased monocyte HLA-DR expression and reduced TNF production in response to LPS, and has proven effective in treating fungal sepsis in chronic granulomatous disease and HIV-associated cryptococcal meningitis (50–52).

Our current findings validate and deepen our previous work on plasma metabolic profiles in ARDS phenotypes (14). While our earlier pilot study identified reduced circulating lipids and elevated glycolytic metabolites in hyperinflammatory ARDS, our present multiomic analysis elucidates the mechanistic underpinnings of these derangements. Mitochondrial stress emerged as a central theme across all mortality-associated MEFISTO factors, with lactoyl amino acids — recently established biomarkers of mitochondrial dysfunction in inherited metabolic disorders and predictors of septic shock mortality — significantly elevated in 3 of the 4 factors (16, 29). Each factor highlighted distinct perturbations in mitochondrial bioenergetics coupled with specific immune signatures: Factor 1 revealed metabolic reprogramming suggestive of the Warburg effect alongside enhanced innate immunity; factor 2 demonstrated specific deficits in fatty acid β-oxidation with impaired immune responses related to liver dysfunction; factor 3 highlighted increased expression of oxidative phosphorylation and electron transport chain genes coupled with interferon program suppression; and factor 5 identified mitochondrial redox imbalance with immune cell proliferation and oxidative stress–induced cellular senescence. The metabolic signatures, together with broad depletion of membrane lipids across all factors, offer mechanistic explanations for the reduced circulating lipids previously observed in our work and independent sepsis cohorts (53, 54). This molecular dissection of ARDS heterogeneity demonstrates the intricate interplay between mitochondrial bioenergetics and immunophenotype, suggesting combination therapies targeting both metabolic derangements and inflammation may achieve synergistic reductions in ARDS and sepsis mortality. Notably, previous experimental work identified mitochondrial dysfunction in alveolar epithelial type 2 cells that was rescued by mitochondrial transfer from mesenchymal stromal cells, resulting in recovered surfactant secretion and reduced lung injury severity, highlighting the therapeutic potential of interventions restoring mitochondrial function (55).

Our phenotype-specific multiomic analyses reveal that within-phenotype biological heterogeneity had modest associations with mortality. Rather, the primary biological differences driving outcome variation were those that distinguish the inflammatory phenotypes from each other. Nevertheless, examination of mortality-associated signatures within each inflammatory phenotype uncovered distinct mechanistic patterns. In hypoinflammatory ARDS, the mortality signature was characterized by profound suppression of translation machinery, suppressed adaptive immunity, and enhanced innate immunity. This signature strongly correlated with moderate-to-high dose of corticosteroid within the preceding 24 hours. Since steroid administration in ROSE was clinician-directed rather than protocol-driven, this association may reflect confounding by indication. Without comprehensive data on steroid dosing and duration, this relationship cannot be interpreted as causal. However, existing evidence suggests patients with hypoinflammatory-like phenotypes may respond poorly to corticosteroids, as demonstrated in a secondary analysis of the VANISH trial, where the hypoinflammatory phenotype experienced worse outcomes when randomized to corticosteroids (56, 57). These findings suggest that steroid responsiveness may vary significantly among ARDS phenotypes, underscoring the necessity for phenotype-stratified clinical trials to optimize therapeutic approaches.

In hyperinflammatory ARDS, mortality was associated with enhanced RUNX1-mediated hematopoietic programs, widespread chromatin remodeling, active cell cycle progression, and oxidative stress–induced senescence. Non-survivors also exhibited HCMV infection pathway enrichment, suggesting viral reactivation, and elevation in long-chain acyl carnitines and lactoyl amino acids, suggestive of mitochondrial metabolic failure. RUNX1 overactivation may be pathogenic, as its knockdown attenuates inflammatory cytokine production in LPS-stimulated macrophages, its inhibition improves survival in septic shock models, and its silencing exosomes ameliorate sepsis-induced acute kidney injury in experimental models (58–60).

Last, metabolomic analyses revealed depleted circulating long-chain PUFAs, likely from oxidative stress–induced peroxidation and consumption of inflammatory lipid mediators, accompanied by elevated plasmalogens and long-chain acyl carnitines indicative of impaired fatty acid β-oxidation. Collectively, these data suggest mortality in the hyperinflammatory phenotype results from multifactorial dysregulation spanning innate and adaptive immunity, platelet activation, lipid metabolism, and estrogen signaling pathways.

Our findings offer several clinical implications. The identification of mortality-associated molecular signatures presents opportunities for targeted interventions based on specific biological mechanisms. These signatures remain stable during the initial 48 hours after ICU admission, providing a potential therapeutic window. Our data indicate multiple contributing pathways to mortality, suggesting combination therapies may yield synergistic benefits, similar to IL-6 inhibitors with dexamethasone in COVID-19–related ARDS (61). Factor 1 represents expansion of immature, immunosuppressive neutrophils characterized by upregulation of emergency granulopoiesis markers (IL1R2, ARG1, CD177, OLFM4), stress response genes (HSPA1A/B, S100A8/9), and tissue-damaging enzymes (MMP8/9), coupled with metabolic hyperactivation (enhanced glycolysis, BCAA metabolism, lipid mediator synthesis), consistent with recent studies showing these populations predict mortality (26). Conversely, factor 2 reflects a metabolically paralyzed state with downregulation of critical antimicrobial peptides (CAMP, DEFA1, LYZ) and defensive molecules (CST3, CFD, BST2) despite slight increases in some granule proteins (MPO, ELANE, PRTN3), alongside disrupted fatty acid metabolism (altered dicarboxylate and monohydroxy fatty acids), impaired protein synthesis responses (EIF2AK4/GCN2), and aberrant GPCR signaling. Genes showing opposing patterns between factors (CYBB, CXCL1, LTF, BPI upregulated in factor 1, downregulated in factor 2) suggest factor 1 cells represent a dysregulated state simultaneously expressing antimicrobial and immunosuppressive markers, while factor 2 demonstrates clear suppression of antimicrobial competence that may prevent effective pathogen clearance (62). Importantly, factor 2 accounted for only 1.5% of the explained transcriptomic variance (vs. 35% for factor 1; Supplemental Table 4), indicating that this transcriptomic signature is a minor contributor to overall outcomes. However, factor 2 accounted for 46% of explained metabolomic variance, suggesting that fatty acid β-oxidation impairment likely represents a broader metabolic dysfunction beyond neutrophils alone, potentially affecting multiple cell types and contributing to the systemic metabolic dysregulation observed in severe sepsis and ARDS. With emerging precision medicine platform trials in critical care and point-of-care phenotyping tools for inflammatory phenotypes, therapeutics targeting these signatures can be systematically evaluated across phenotypes (13, 63).

Our study has several key strengths that enhance the robustness and generalizability of its findings. To our knowledge, our analysis, which used samples from 160 patients in the multicenter ROSE trial, represents the largest multiomics analysis in ARDS to date. Compared with our previous pilot metabolomic investigation, which analyzed a small, selected subset at a single time point, the current study employed systematic biospecimen collection with longitudinal sampling and adequate statistical power, detecting substantially greater biological diversity and enabling robust phenotypic comparisons. The clinical trial framework ensured standardized care and systematic biospecimen collection, minimizing treatment-related confounding. Our multimodal approach provides important insights into cellular programming and systemic metabolism in ARDS and sepsis. The 2–time point design established signature stability, critical for therapeutic target identification. External validation in EARLI, a diverse sepsis cohort that captures patients early in critical illness, demonstrates these molecular signatures represent generalizable biological states rather than ARDS-specific findings. This cross-syndrome reproducibility strengthens clinical applicability, as therapeutic interventions targeting these signatures could benefit the broader population of critically ill patients with sepsis who share similar molecular phenotypes, aligning with evidence that ARDS inflammatory phenotypes extend to sepsis (24) and overlap with other protein and transcriptional subtypes (49, 57, 64, 65).

Important limitations include the inability of observational human biospecimen data to establish causality between identified signatures and outcomes. Whole-blood transcriptomics precludes attribution of gene expression patterns to specific immune cell populations. While we employed computational deconvolution using CIBERSORTx to estimate cell type contributions, this approach has inherent limitations, including dependence on reference dataset selection, inability to capture disease-specific or potentially novel cell states, and potential confounding by shared gene expression programs across cell types. Furthermore, transcriptional programs may not reflect functional protein capacity, particularly in contexts such as emergency granulopoiesis, where gene expression patterns can be developmentally regulated independently of protein translation. Similarly, untargeted metabolomics provides limited source information for the observed differences in circulation, which may include liver, kidney, and lung. This multitissue origin represents both a limitation (we cannot definitively attribute metabolic changes to specific cell types) and a strength (circulating metabolites constitute the metabolic environment shaping immune cell function). The absence of comprehensive pathogen data restricts contextualizing these molecular signatures within the broader pathophysiology of ARDS and sepsis. The pronounced mortality difference between phenotypes in our cohort (24% vs. 61%) may have enhanced detection of certain signatures, particularly factor 5, which explained minimal model variance and may not retain its mortality association in cohorts with smaller phenotype differences. Finally, clinical utility of these molecular signatures requires further investigation in both experimental models and clinical studies.

In conclusion, this comprehensive multiomic analysis reveals insights into the molecular heterogeneity of ARDS and sepsis. Inflammatory phenotypes of ARDS and sepsis reflect distinct biological processes with profound differences in mitochondrial function, immune response, and metabolic regulation. Mortality-associated molecular states suggest complex interplay between phenotype-specific and phenotype-independent pathways affecting patient outcomes. Future studies must determine tissue origins of these circulating signatures, the impact of specific pathogens, and test viable therapeutic targets in experimental models, laying groundwork for interventions that address the molecular complexity of critical illness.

## Methods

### Study design and cohorts

#### Sex as a biological variable.

Our study examined male and female participants. Sex was included as a covariate in regression analyses.

#### Primary cohort.

The ROSE randomized trial of neuromuscular blockade for moderate-to-severe ARDS enrolled 1,006 patients from January 2016 to April 2018 (15). Patients were randomized to continuous cisatracurium infusion with deep sedation versus usual care, with the trial stopping early due to futility for the primary outcome of 90-day mortality. LCA of clinical and protein biomarker data was previously performed on all patients with day 0 biospecimens available, with participants assigned probabilities of membership to hyper- or hypo-inflammatory phenotypes (10). We randomly selected 80 patients from each phenotype who had a >0.9 probability of phenotype membership. This sample size was determined a priori to enable detection of differences between survivors and non-survivors within each phenotype, assuming mortality rates of 40% in hyperinflammatory and 20% in hypoinflammatory ARDS based on prior studies (5, 6, 8, 9, 11). Using the *MetSizeR* package with probabilistic principal components analysis and a fixed FDR of 0.05, this sample size (32 predicted deaths in hyperinflammatory and 16 in hypoinflammatory) provided more than 90% power to detect metabolic differences via untargeted profiling (27, 66, 67). Samples were obtained from the NIH National Heart, Lung, and Blood Institute (NHLBI) biorepository, BioLINCC.

#### Validation cohort.

The Early Acute Renal and Lung Injury (EARLI) study is an ongoing prospective observational cohort of critically ill adults admitted to ICUs at the UCSF Moffitt-Long Hospital and Zuckerberg San Francisco General Hospital. Patients are eligible upon ICU admission from the emergency room, excluding those with isolated neurological/neurosurgical indications or trauma service admissions. From this cohort, we analyzed 3 partially overlapping subgroups (Supplemental Figure 3): 195 patients with sepsis (2008 through 2016) who previously underwent metabolic profiling (27), 196 participants with hypotension or requiring invasive mechanical ventilation in the emergency room and sepsis (2010 through 2018) who previously underwent transcriptomic profiling (28), and 308 patients from 818 patients with sepsis (2008 through 2019) who underwent LCA of clinical and protein biomarker data (24). This subset of 308 patients was selected because they had both LCA phenotype designation and transcriptomic data, metabolomic data, or both available. Sepsis diagnosis was adjudicated through retrospective physician review of electronic medical records using Sepsis-2 criteria, incorporating all available clinical and microbiologic data while blinded to phenotype or biological profiling data (68). Patients whose initial sepsis diagnosis occurred more than 2 days after ICU admission were excluded. We analyzed lactate values from 546 of 818 phenotyped patients with sepsis in EARLI who had clinical lactate measurements at days 0–2 of enrollment, including subsequent values for longitudinal comparisons.

### Biomarker measurements

#### Metabolic profiling.

EDTA plasma (150 μL) from day 0 and day 2 of ROSE trial enrollment was batch-shipped to Metabolon, was precipitated with methanol, and underwent untargeted metabolic profiling using 3 complementary methods: reverse-phase chromatography/ultra performance liquid chromatography-tandem mass spectrometry (RP/UPLC-MS/MS) with positive electrospray ionization (ESI), RP/UPLC-MS/MS with negative ESI, and hydrophilic interaction liquid chromatography/UPLC-MS/MS with negative ESI. Metabolon performed peak identification using an in-house library in 2023, as well as quality control and batch normalization.

In EARLI, 150 μL of citrated plasma underwent identical untargeted profiling methodology, with peaks identified using Metabolon’s in-house library in 2017 (27).

#### RNA sequencing.

In the ROSE cohort, whole-blood samples from day 0 and day 2 of trial enrollment were collected in PAXgene tubes, were stored at –80°C, and had RNA extracted using QIAGEN RNeasy kit followed by DNase treatment as previously described (10). In EARLI, whole-blood RNA sequencing was performed using a similar methodology (28).

### Statistics

Analyses were conducted in R version 4.3.2. Clinical variables and demographics were compared between the phenotypes using Welch’s *t* test, Wilcoxon’s rank-sum test, χ^2^ test, or Fisher’s exact test as appropriate based on variable type, distribution, and expected frequency. A *P* value less than 0.05 was considered significant.

For metabolomic analyses, unknown metabolites and those with >25% missingness in both phenotypes were removed. Following Kokla et al.’s approach to minimize imputation error (69), metabolites with >25% missingness in either phenotype were imputed using a uniform distribution ranging from ½ minimum to minimum observed value of the metabolite across all samples. The remaining metabolites were imputed using Random Forest (*missForest*). Metabolic profiles were compared via differential abundance analysis using limma (*MetaboAnalystR* package), adjusting for age, sex, BMI, relevant medications (propofol, dexmedetomidine, corticosteroids), comorbid liver disease, and GFR (70). For day 2 analyses, randomization arm was added as a covariate, as samples were obtained after the administration of trial agents. Metabolite enrichment analysis was performed using ChemRICH (71), a chemical similarity-based statistical enrichment approach that overcomes limitations of traditional pathway analysis. By grouping metabolites based on chemical ontologies and structural similarity, ChemRICH generates study-specific, non-overlapping metabolite sets with self-contained enrichment statistics independent of background database size. For our analysis, differentially abundant metabolites at each time point (day 0 or day 2) with their identifiers (SMILES, InChIKeys, PubChem IDs) and Metabolon class annotations were selected. After resolving duplicate entries and completing missing PubChem IDs through database searches, the dataset was processed through the ChemRICH web interface.

For each metabolite, we constructed linear mixed effects models to analyze changes in metabolite values over time based on 90-day mortality outcome. The primary model included fixed effects for time, mortality, treatment arm, age, sex, and BMI, with a random intercept for each participant. We tested the significance of the time-by-mortality interaction by comparing this model and a null model without the interaction term using likelihood ratio tests. The coefficient of the time-by-mortality interaction represents the differential trajectory of metabolite levels between survivors and non-survivors from day 0 to day 2, with positive values indicating greater increases (or smaller decreases) in non-survivors. *P* values from model comparisons (FDR < 0.05) were used to assess statistical significance of these differential trajectories. For analysis of metabolic class trajectories over time, we annotated differentially expressed metabolites with pathway information from Metabolon’s database. Fold-changes were calculated by exponentiating the model coefficients and adding 1, representing the relative change in metabolite levels between survivors and non-survivors. We performed enrichment analysis using ChemRICH as described above. Significantly enriched pathways (FDR < 0.05) were classified as increased or decreased based on the proportion (>0.5) of increased metabolites within each pathway.

For multiomics analyses in the full study cohort, we implemented a rigorous filtration pipeline to select only the most abundant and variable analytes, thereby avoiding imputation that can introduce artifacts in integrated multiomics analyses. As such, unknown metabolites and xenobiotics were removed. Metabolites with >10% missingness were removed. Remaining metabolites underwent log transformation, quantile normalization, and selection of the top 500 by median absolute deviation (MAD), followed by *z*-scaling. Transcriptomic data underwent variance-stabilizing transformation, with the top 2,500 genes selected by MAD and subsequently *z*-scaled. For each patient, day 0 and day 2 metabolite and gene expression data were entered into a MEFISTO model (*mofa2* package) (25). MEFISTO is an unsupervised, multimodal, temporally informed dimensionality reduction tool to identify predominant patterns of variation in omics data. MEFISTO extends conventional matrix factorization by incorporating a functional view on latent factors based on Gaussian processes, allowing for modeling of temporal relationships in the data. Our implementation treated the entire patient cohort as a single group while declaring time as a covariate, facilitating joint decomposition of multiomics data matrices into latent factors (Z) with corresponding feature weights (W), with temporal structure modeled through a squared exponential covariance function. This framework allowed for identification of both smooth (time-dependent) and non-smooth (time-independent) variation patterns, providing insights into temporal dynamics of molecular responses in ARDS patients while accounting for cohort level heterogeneity. After model fitting, the resulting factor values (*Z*) were extracted to quantify the strength of each identified molecular covariation pattern for each patient at each time point, allowing us to characterize the temporal dynamics of metabolomic and transcriptomic responses in ARDS.

We selected 10 latent factors for initial analysis. The total variance (*R*^2^) explained for each data modality and per factor was calculated to determine the primary sources of dataset heterogeneity. To determine the association of MEFISTO factors with clinical variables, we performed linear regression for categorical predictors (with factor value as the outcome) and Spearman’s correlation for continuous predictors using day 0 factor values. Clinical variables with missingness were left as missing (not imputed). FDR-adjusted *P* < 0.05 was considered significant. Gene set enrichment analysis was performed on latent MEFISTO factors using Reactome and MitoCarta 3.0 gene sets, while a metabolite set was generated using Metabolon’s annotated library (72, 73). To test for interaction between MEFISTO factors and time regarding mortality, we implemented linear mixed effects regression models with 90-day mortality, time point, and their interaction as fixed effects, including a random intercept for each patient to account for within-patient correlation in measurements over time.

For multiomics analyses within each LCA phenotype, the same data processing pipeline was applied with MAD-based selection of metabolites and gene transcripts performed within each phenotype.

To assess the relationship between inferred cell type composition and sample-level factors for factors 1 and 2, we computed Spearman’s rank correlations between cell type proportions and factor values across samples. Cell type proportions were estimated using CIBERSORTx, leveraging the reference generated by Kwok et al. (26, 74). Spearman’s correlation coefficients were calculated to identify significant associations between specific cell individual and aggregated populations of interest and factors 1 and 2.

For validation studies in EARLI, the same pipeline was applied to prepare metabolite and transcriptomic data. The relative weights of the top 100 features within each MEFISTO latent factor that were present in EARLI were used to calculate factor values for each EARLI patient. Specifically, factor values were calculated as the weighted sum of normalized feature measurements, using weights derived from our original MEFISTO model. Associations between factor values per patient and clinical outcomes were tested using Wilcoxon’s rank-sum tests.

All analyses were adjusted for multiple comparisons using the Benjamini-Hochberg FDR with significance set at FDR < 0.05. Continuous data are shown as mean ± standard deviation and median (IQR) based on distribution, and categorical data are shown as *N* (%).

### Study approval

Informed consent was obtained from patients or their surrogates, except when the IRB approved a waiver of consent for the following circumstances: patients who remained too critically ill to provide consent themselves and for whom no surrogate decision-maker could be identified within 28 days of hospital discharge and patients who died prior to consent being obtained from either the patient or their surrogate. The Institutional Review Board of the UCSF approved the enrollment of humans in the EARLI observational cohort and the ROSE randomized controlled trial.

### Data availability

Supporting values for all the manuscript and supplemental figures, including complete results of gene and metabolite set enrichment analyses, are provided in the Supporting Data Values file. Transcriptomic data for the ROSE trial participants are available at dbGAP: https://www.ncbi.nlm.nih.gov/projects/gap/cgi-bin/study.cgi?study_id=phs003929.v1.p1; dbGaP Study Accession phs003929.v1.p1. Metabolomic data have been deposited in the NIH Metabolomics Workbench (uploaded October 16, 2025; https://doi.org/10.21228/M8ZV9M) (75). Clinical data and biospecimens from the ROSE trial are available through the NHLBI BioLINCC repository (https://biolincc.nhlbi.nih.gov/studies/) to qualified researchers upon request and completion of appropriate data use agreements. The code for the analyses performed in this manuscript is available on GitHub: https://github.com/nalipan1/rose_manuscript, commit ID 8b3859e.

## Author contributions

NAL, LN, and CSC contributed to the conceptualization of this work. NAL, LN, AS, and CSC developed the methodology. NAL and LN conducted the investigation and created the visualizations. NAL and CSC acquired funding for the project. Project administration was performed by NAL, LN, C Leroux, KB, SAC, OC, SC, TH, CH, KK, CRL, DL, C Lin, KL, LM, AR, AS, ES, NS, KAS, MFW, AW, HZ, MAM, and CSC. NAL and CSC provided supervision. NAL, LN, and CSC wrote the original draft. NAL, LN, PS, C Leroux, KB, SAC, OC, SC, TH, CH, KK, CRL, DL, C Lin, KL, LM, AR, AS, ES, NS, KS, MFW, AW, HZ, AJR, KAS, MAM, and CSC reviewed and edited the manuscript.

NAL and LN are co–first authors. The order of names was determined based on NAL driving the study concept, conducting the metabolomics analyses, and contributing to the biological interpretation, while LN led the transcriptomic analyses and designed the computational approach. Both authors made substantial and essential contributions to the work.

## Funding support

This work is the result of NIH funding, in whole or in part, and is subject to the NIH Public Access Policy. Through acceptance of this federal funding, the NIH has been given a right to make the work publicly available in PubMed Central.

- NIH funding (National Heart Lung and Blood Institute [NHLBI] grants K23HL173669 [to NAL], R35HL140026, and R35177135 [to CSC]).
- NIH funding (National Institute of General Medical Sciences grant R35GM136312 [to KAS]).

## Supplementary Material

Supplemental data

ICMJE disclosure forms

Supporting data values

## Version 1 12/02/2025

In-Press Preview

## Version 2 02/02/2026

Electronic publication

## References

[1] Bellani G (2016). Epidemiology, patterns of care, and mortality for patients with acute respiratory distress syndrome in intensive care units in 50 countries. JAMA.

[2] Rudd KE (2020). Global, regional, and national sepsis incidence and mortality, 1990–2017: analysis for the Global Burden of Disease Study. Lancet.

[3] Marshall JC (2014). Why have clinical trials in sepsis failed?. Trends Mol Med.

[4] Matthay MA (2017). Clinical trials in acute respiratory distress syndrome: challenges and opportunities. Lancet Respir Med.

[5] Calfee C (2014). Subphenotypes in acuterespiratory distress syndrome: latent class analysis of data from two randomised controlled trials. Lancet Respir Med.

[6] Calfee C (2018). Acute respiratory distress syndrome subphenotypes and differential response to simvastatin: secondary analysis of a randomised controlled trial. Lancet Respir Med.

[7] Sinha P (2020). Prevalence of phenotypes of acute respiratory distress syndrome in critically ill patients with COVID-19: a prospective observational study. Lancet Respir Med.

[8] Sinha P (2022). Latent class analysis-derived subphenotypes are generalisable to observational cohorts of acute respiratory distress syndrome: a prospective study. Thorax.

[9] Sinha P (2018). Latent class analysis of ARDS subphenotypes: a secondary analysis of the statins for acutely injured lungs from sepsis (SAILS) study. Intensive Care Med.

[10] Sinha P (2024). Molecular phenotypes of acute respiratory distress syndrome in the ROSE trial have differential outcomes and gene expression patterns that differ at baseline and longitudinally over time. Am J Respir Crit Care Med.

[11] Famous K (2017). Acute respiratory distress syndrome subphenotypes respond differently to randomized fluid management strategy. Am J Respir Crit Care Med.

[12] Delucchi K (2018). Stability of ARDS subphenotypes over time in two randomised controlled trials. Thorax.

[14] Alipanah-Lechner N (2023). Plasma metabolic profiling implicates dysregulated lipid metabolism and glycolytic shift in hyperinflammatory ARDS. Am J Physiol Lung Cell Mol Physiol.

[15] Moss M (2019). Early neuromuscular blockade in the acute respiratory distress syndrome. Reply. N Engl J Med.

[16] Sharma R (2021). Circulating markers of NADH-reductive stress correlate with mitochondrial disease severity. J Clin Invest.

[17] Levy B (2005). Bench-to-bedside review: is there a place for epinephrine in septic shock?. Crit Care.

[18] Day NP (1996). The effects of dopamine and adrenaline infusions on acid-base balance and systemic haemodynamics in severe infection. Lancet.

[19] Suomalainen A (2011). FGF-21 as a biomarker for muscle-manifesting mitochondrial respiratory chain deficiencies: a diagnostic study. Lancet Neurol.

[20] Debray FG (2007). Diagnostic accuracy of blood lactate-to-pyruvate molar ratio in the differential diagnosis of congenital lactic acidosis. Clin Chem.

[21] Kemperman RH (2023). B-169 Beta-hydroxybutyrate/acetoacetate ratio as indicator for mitochondrial diseases utilizing a novel LC-MS/MS based ketone body panel. Clin Chem.

[22] Martinez-Reyes I, Chandel NS (2020). Mitochondrial TCA cycle metabolites control physiology and disease. Nat Commun.

[23] Li X (2022). Lactate metabolism in human health and disease. Signal Transduct Target Ther.

[24] Sinha P (2023). Identifying molecular phenotypes in sepsis: an analysis of two prospective observational cohorts and secondary analysis of two randomised controlled trials. Lancet Respir Med.

[25] Velten B (2022). Identifying temporal and spatial patterns of variation from multimodal data using MEFISTO. Nat Methods.

[26] Kwok AJ (2023). Neutrophils and emergency granulopoiesis drive immune suppression and an extreme response endotype during sepsis. Nat Immunol.

[27] Rogers AJ (2021). Plasma metabolites in early sepsis identify distinct clusters defined by plasma lipids. Crit Care Explor.

[28] Kalantar KL (2022). Integrated host-microbe plasma metagenomics for sepsis diagnosis in a prospective cohort of critically ill adults. Nat Microbiol.

[29] Rogers RS (2024). Circulating N-lactoyl-amino acids and N-formyl-methionine reflect mitochondrial dysfunction and predict mortality in septic shock. Metabolomics.

[30] Ahl PJ (2020). Met-Flow, a strategy for single-cell metabolic analysis highlights dynamic changes in immune subpopulations. Commun Biol.

[31] Stienstra R (2017). Specific and complex reprogramming of cellular metabolism in myeloid cells during innate immune responses. Cell Metab.

[32] Davies LC (2019). Diversity and environmental adaptation of phagocytic cell metabolism. J Leukoc Biol.

[33] Raghuraman S (2016). The emerging role of epigenetics in inflammation and immunometabolism. Trends Endocrinol Metab.

[34] Ratter JM (2018). Environmental signals influencing myeloid cell metabolism and function in diabetes. Trends Endocrinol Metab.

[35] Gaber T (2017). Metabolic regulation of inflammation. Nat Rev Rheumatol.

[36] Marelli-Berg FM, Jangani M (2018). Metabolic regulation of leukocyte motility and migration. J Leukoc Biol.

[37] Ratter JM (2018). In vitro and in vivo effects of lactate on metabolism and cytokine production of human primary PBMCs and monocytes. Front Immunol.

[38] Christensen E (1991). Omega-oxidation of fatty acids studied in isolated liver cells. Biochim Biophys Acta.

[39] Goetzman ES (2024). Dietary dicarboxylic acids provide a nonstorable alternative fat source that protects mice against obesity. J Clin Invest.

[40] Kumps A (2002). Metabolic, nutritional, iatrogenic, and artifactual sources of urinary organic acids: a comprehensive table. Clin Chem.

[41] Tonsgard JH, Getz GS (1985). Effect of Reye’s syndrome serum on isolated chinchilla liver mitochondria. J Clin Invest.

[42] Ranea-Robles P, Houten SM (2023). The biochemistry and physiology of long-chain dicarboxylic acid metabolism. Biochem J.

[43] (2021). Plasmalogens and chronic inflammatory diseases. Front Physiol.

[44] Morel J (2017). Simvastatin pre-treatment improves survival and mitochondrial function in a 3-day fluid-resuscitated rat model of sepsis. Clin Sci (Lond).

[45] Jones AE (2018). Effect of levocarnitine vs placebo as an adjunctive treatment for septic shock: the Rapid Administration of Carnitine in Sepsis (RACE) randomized clinical trial. JAMA Netw Open.

[46] Hadjadj J (2020). Impaired type I interferon activity and inflammatory responses in severe COVID-19 patients. Science.

[47] Heinonen S (2020). Immune profiles provide insights into respiratory syncytial virus disease severity in young children. Sci Transl Med.

[48] Bost P (2020). Host-viral infection maps reveal signatures of severe COVID-19 patients. Cell.

[49] Scicluna BP (2017). Classification of patients with sepsis according to blood genomic endotype: a prospective cohort study. Lancet Respir Med.

[50] Docke WD (1997). Monocyte deactivation in septic patients: restoration by IFN-gamma treatment. Nat Med.

[51] Marciano BE (2004). Long-term interferon-gamma therapy for patients with chronic granulomatous disease. Clin Infect Dis.

[52] Jarvis JN (2012). Adjunctive interferon-γ immunotherapy for the treatment of HIV-associated cryptococcal meningitis: a randomized controlled trial. AIDS.

[53] Chouchane O (2024). The plasma lipidomic landscape in patients with sepsis due to community-acquired pneumonia. Am J Respir Crit Care Med.

[54] McCann MR (2025). Early sepsis metabolic changes in kidney and liver precede clinical evidence of organ dysfunction. Am J Respir Cell Mol Biol.

[55] Islam MN (2012). Mitochondrial transfer from bone-marrow-derived stromal cells to pulmonary alveoli protects against acute lung injury. Nat Med.

[56] Antcliffe DB (2019). Transcriptomic signatures in sepsis and a differential response to steroids. From the VANISH randomized trial. Am J Respir Crit Care Med.

[57] Neyton LPA (2024). Host and microbe blood metagenomics reveals key pathways characterizing critical illness phenotypes. Am J Respir Crit Care Med.

[58] Cunningham L (2012). Identification of benzodiazepine Ro5-3335 as an inhibitor of CBF leukemia through quantitative high throughput screen against RUNX1-CBFβ interaction. Proc Natl Acad Sci U S A.

[59] Luo MC (2016). Runt-related transcription factor 1 (RUNX1) binds to p50 in macrophages and enhances TLR4-triggered inflammation and septic shock. J Biol Chem.

[60] Zhang Y (2021). Endothelial progenitor cells-derived exosomal microRNA-21-5p alleviates sepsis-induced acute kidney injury by inhibiting RUNX1 expression. Cell Death Dis.

[61] Zeraatkar D (2022). Use of tocilizumab and sarilumab alone or in combination with corticosteroids for covid-19: systematic review and network meta-analysis. BMJ Med.

[62] Pham L (2022). Neutrophil trafficking to the site of infection requires Cpt1a-dependent fatty acid β-oxidation. Commun Biol.

[63] https://clinicaltrials.gov/study/NCT04009330.

[64] Bos LDJ (2019). Understanding heterogeneity in biologic phenotypes of acute respiratory distress syndrome by leukocyte expression profiles. Am J Respir Crit Care Med.

[65] Davenport EE (2016). Genomic landscape of the individual host response and outcomes in sepsis: a prospective cohort study. Lancet Respir Med.

[66] https://CRAN.R-project.org/package=MetSizeR.

[67] Nyamundanda G (2013). MetSizeR: selecting the optimal sample size for metabolomic studies using an analysis based approach. BMC Bioinformatics.

[69] Kokla M (2019). Random forest-based imputation outperforms other methods for imputing LC-MS metabolomics data: a comparative study. BMC Bioinformatics.

[70] Pang Z (2024). MetaboAnalystR 4.0: a unified LC-MS workflow for global metabolomics. Nat Commun.

[71] Barupal DK, Fiehn O (2017). Chemical similarity enrichment analysis (ChemRICH) as alternative to biochemical pathway mapping for metabolomic datasets. Sci Rep.

[72] Rath S (2021). MitoCarta3.0: an updated mitochondrial proteome now with sub-organelle localization and pathway annotations. Nucleic Acids Res.

[73] Milacic M (2024). The Reactome Pathway Knowledgebase 2024. Nucleic Acids Res.

[74] Newman AM (2019). Determining cell type abundance and expression from bulk tissues with digital cytometry. Nat Biotechnol.

[75] Sud M (2016). Metabolomics Workbench: an international repository for metabolomics data and metadata, metabolite standards, protocols, tutorials and training, and analysis tools. Nucleic Acids Res.

