# Supplementary material for: Longitudinal multiomic signatures of ARDS and sepsis inflammatory phenotypes identify pathways associated with mortality
Source: J Clin Invest. 2025 Dec 2;136(3):e196290. doi: 10.1172/JCI196290 (PMC12867137; doi:10.1172/JCI196290)
Supplement: Supplemental data [file jci-136-196290-s009.pdf]

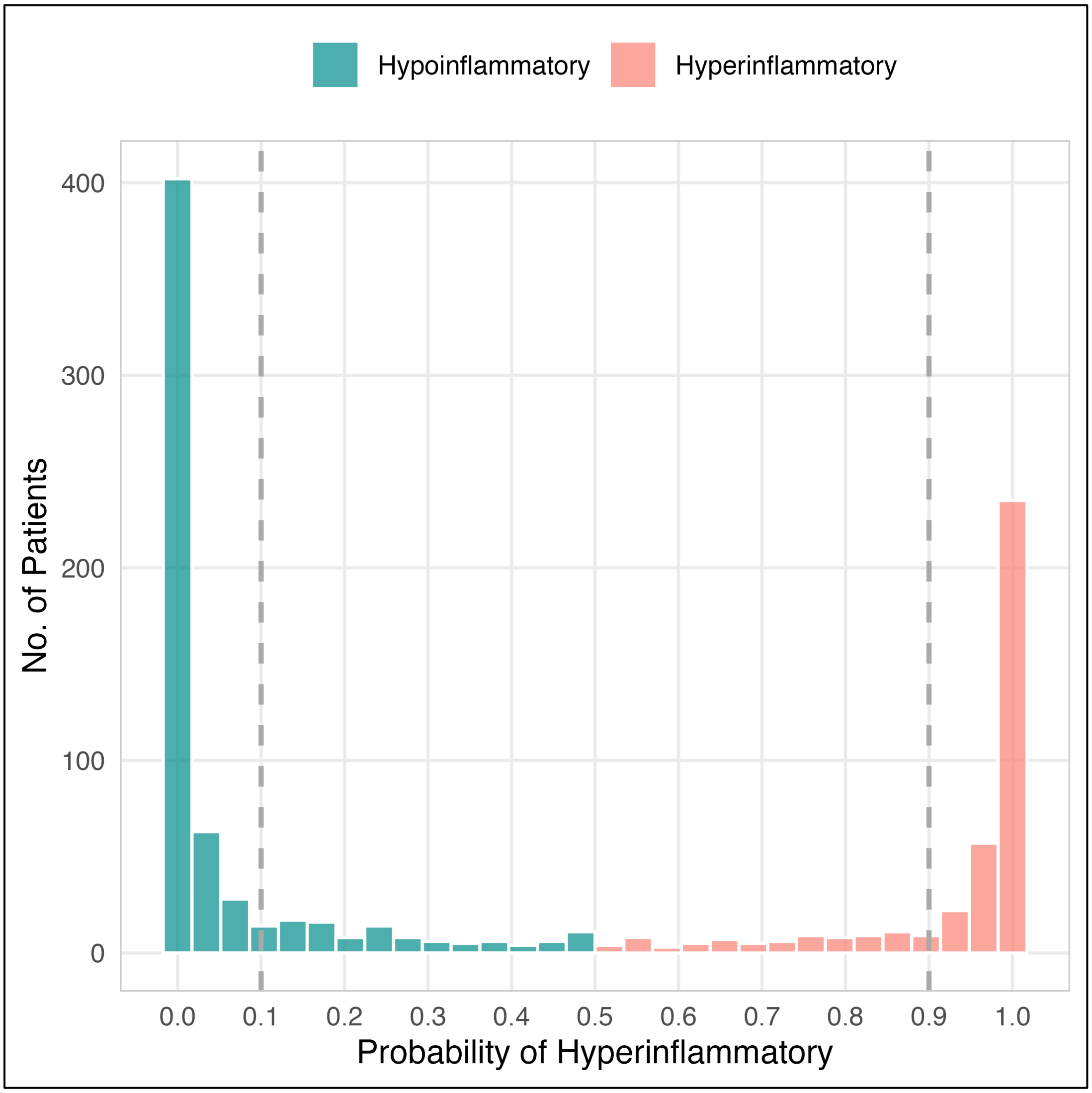

Supplemental Figure 1. Distribution of phenotype probabilities from latent class analysis of 1006 patients in the ROSE trial. Vertical dashed lines indicate the 0.1 and 0.9 thresholds, highlighting regions of high classification certainty into the Hyperinflammatory or Hypoinflammatory phenotypes.

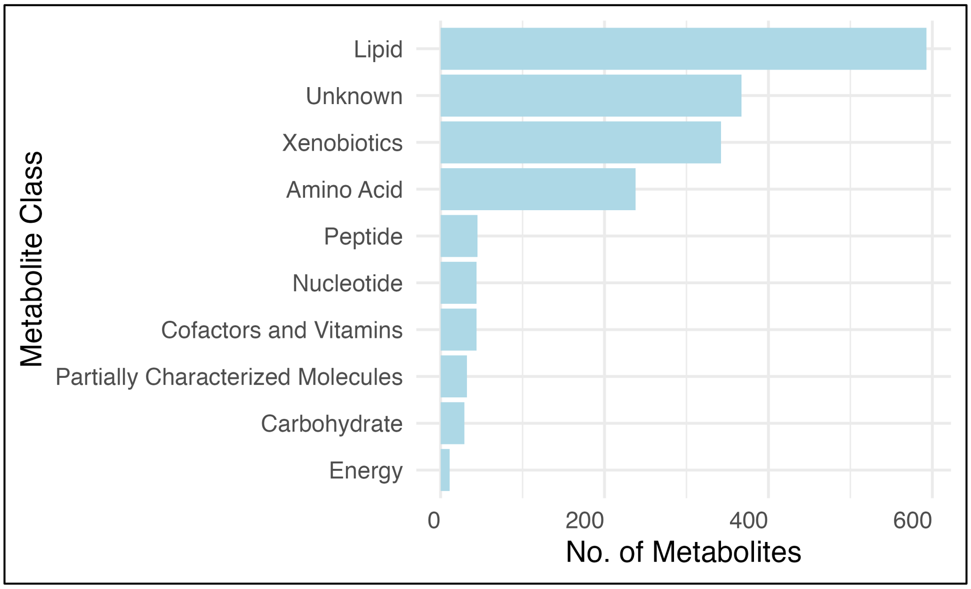

Supplemental Figure 2. Results of untargeted metabolic profiling of EDTA plasma from 160 ROSE trial participants.

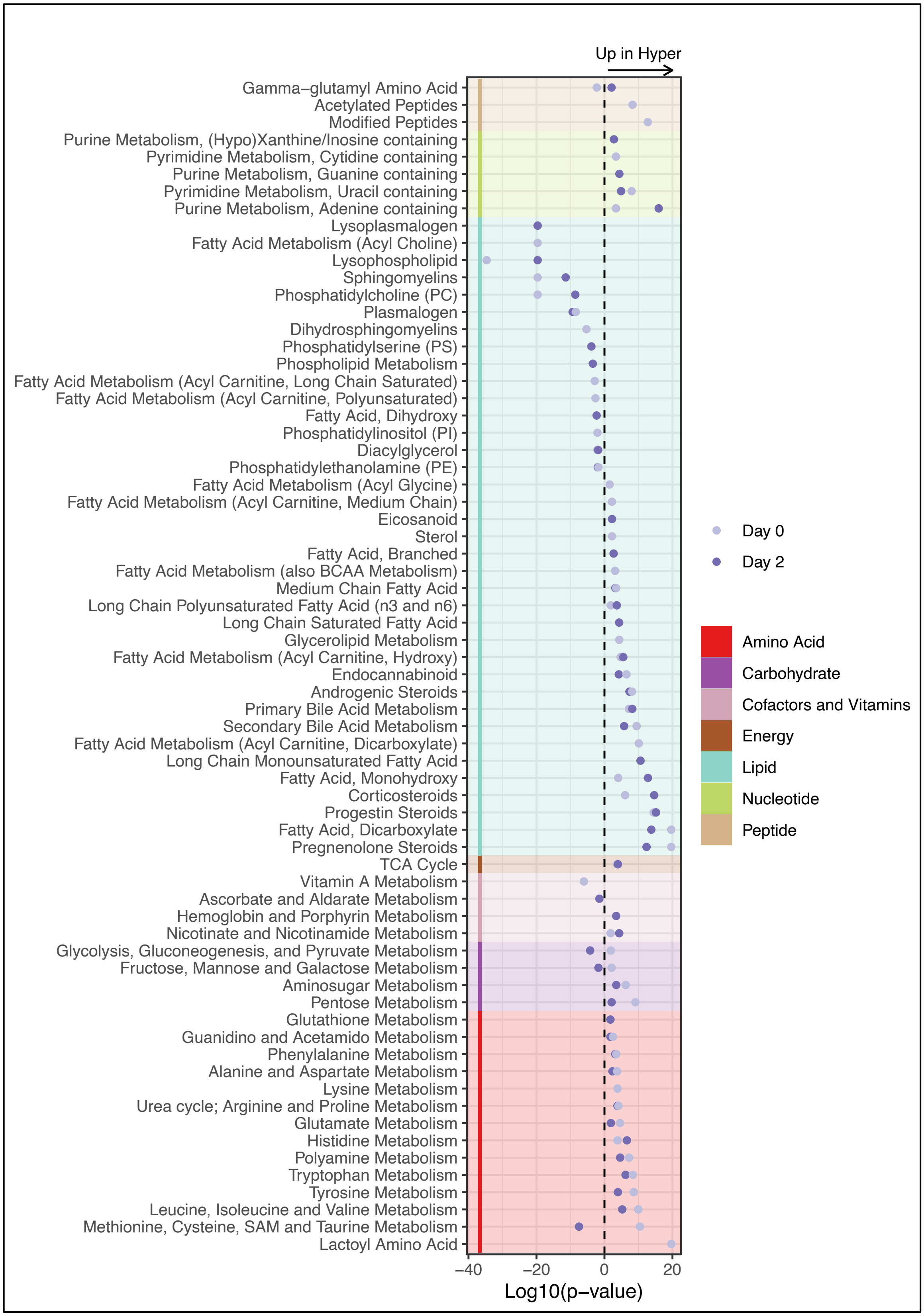

Supplemental Figure 3. Enrichment analysis of differentially abundant metabolites by LCA phenotype in the ROSE cohort. Only significant pathways are displayed (FDR <0.05). Xenobiotics and partially characterized molecules are not depicted.

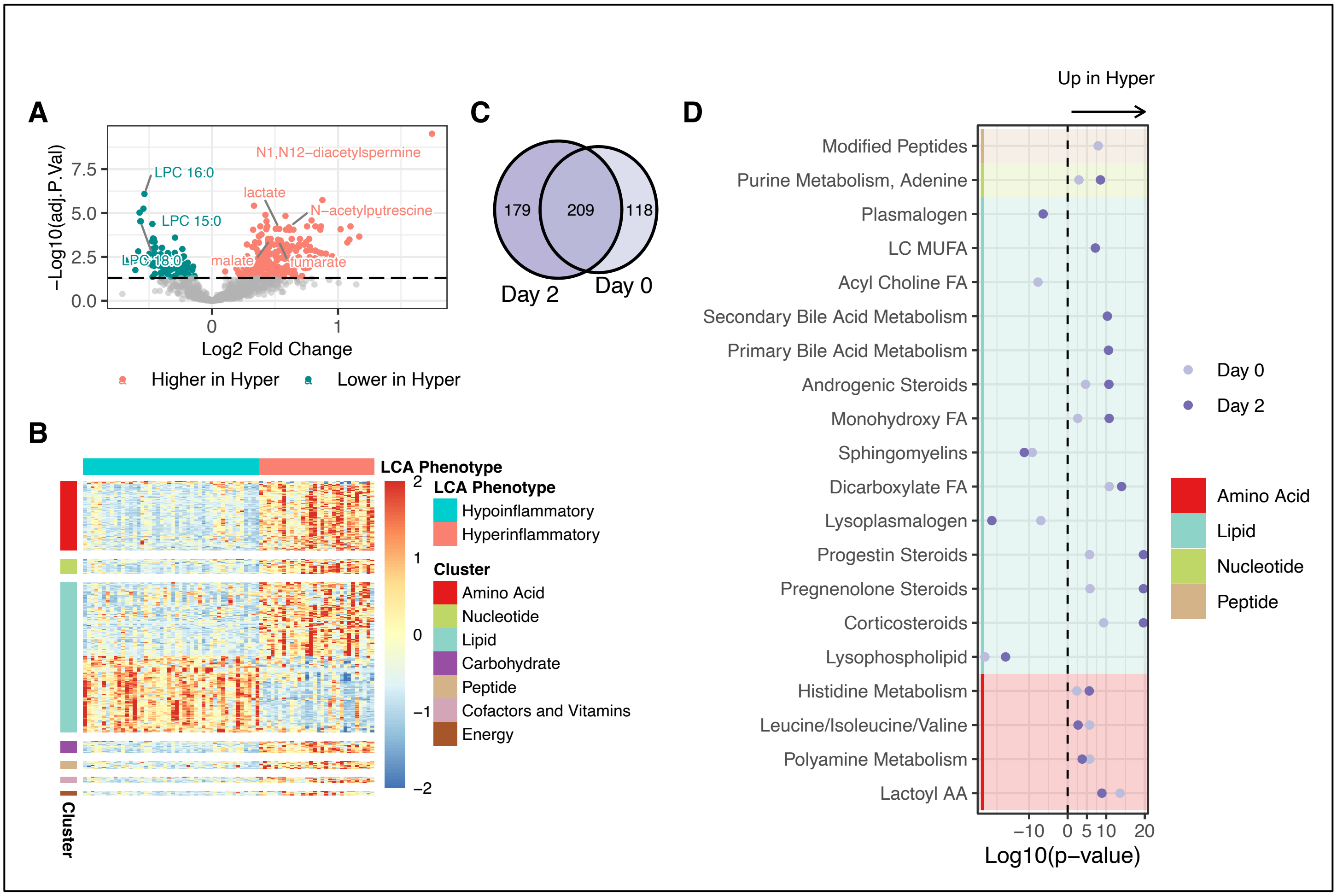

Supplemental Figure 4. Metabolic profiling of latent class analysis (LCA) phenotypes in patients with pneumonia as the primary risk factor for ARDS (N = 85), sensitivity analysis for vasopressor use. (**A**) Volcano plot showing differentially abundant metabolites between Hyperinflammatory and Hypoinflammatory ARDS at Day 0, determined by limma adjusted for covariates (age, sex, BMI, medications, liver disease, GFR, and vasopressor use at study enrollment). (**B**) Heatmap of differentially abundant metabolites by LCA phenotype at Day 0 as determined by limma with adjustment for aforementioned covariates. Z-scaled log-transformed metabolite intensities are grouped by phenotype. (**C**) Venn diagram showing overlap of differentially abundant metabolites at Day 0 and Day 2 (Day 2 also adjusted for randomization arm). (**D**) Metabolite pathway enrichment analysis comparing Hyperinflammatory vs Hypoinflammatory groups at Day 0 and Day 2. X-axis shows signed log10(p-value), with positive values indicating positive enrichment in Hyperinflammatory group and negative values indicating positive enrichment in Hypoinflammatory group. Top 20 significant pathways are shown. AA = amino acid; FA = fatty acid; LC = long chain; MUFA = monounsaturated fatty acid

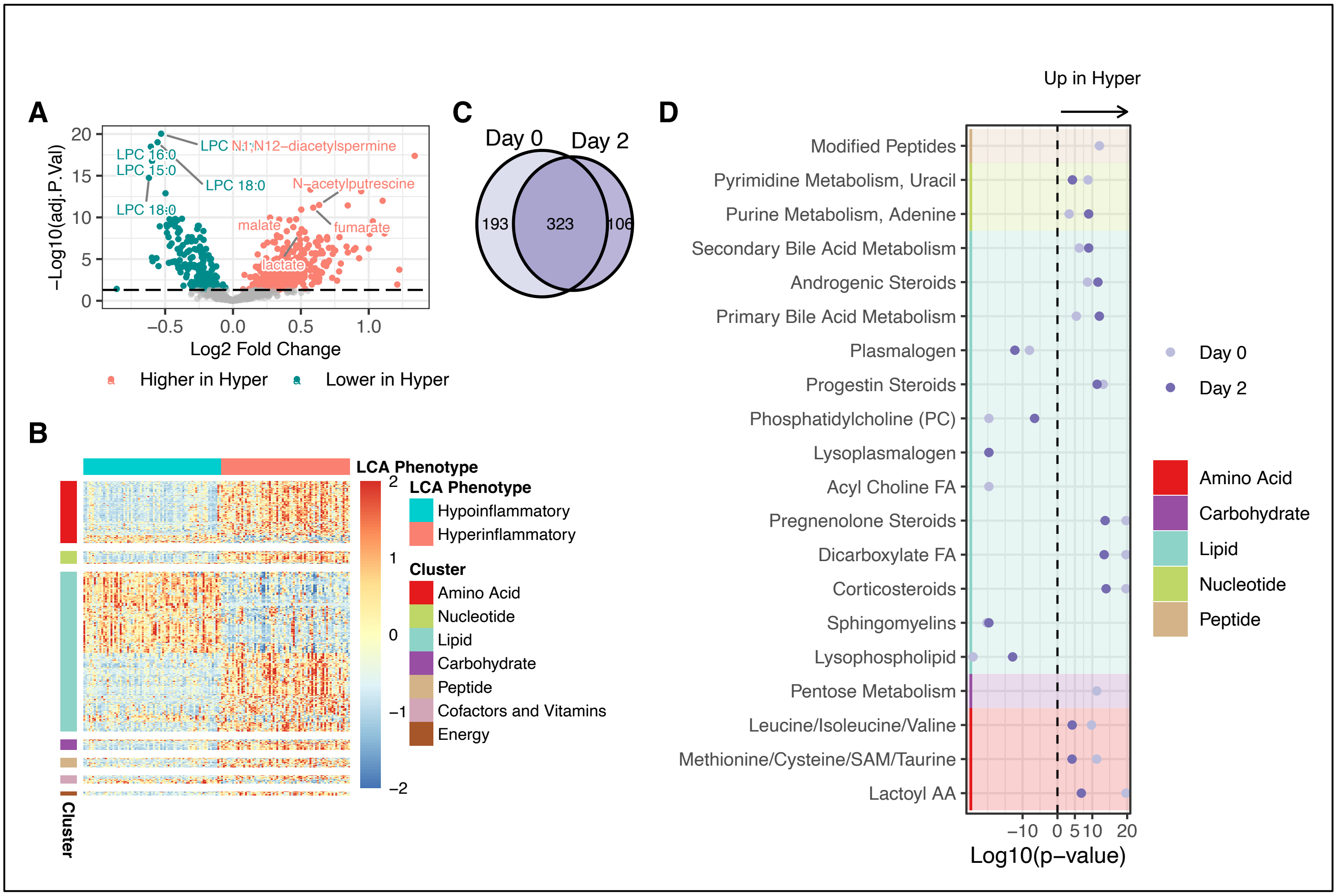

Supplemental Figure 5. Metabolic profiling of latent class analysis (LCA) phenotypes sensitivity analysis for renal replacement therapy. (**A**) Volcano plot showing differentially abundant metabolites between Hyperinflammatory and Hypoinflammatory ARDS at Day 0, determined by limma adjusted for covariates (age, sex, BMI, medications, liver disease, GFR, and renal replacement therapy at Day 0). (**B**) Heatmap of differentially abundant metabolites by LCA phenotype at Day 0 as determined by limma with adjustment for aforementioned covariates. Z-scaled log-transformed metabolite intensities are grouped by phenotype. (**C**) Venn diagram showing overlap of differentially abundant metabolites at Day 0 and Day 2 (Day 2 also adjusted for randomization arm). (**D**) Metabolite pathway enrichment analysis comparing Hyperinflammatory vs Hypoinflammatory groups at Day 0 and Day 2. X-axis shows signed log10(p-value), with positive values indicating positive enrichment in Hyperinflammatory group and negative values indicating positive enrichment in Hypoinflammatory group. Top 20 significant pathways are shown. AA = amino acid; FA = fatty acid; LC = long chain; MUFA = monounsaturated fatty acid

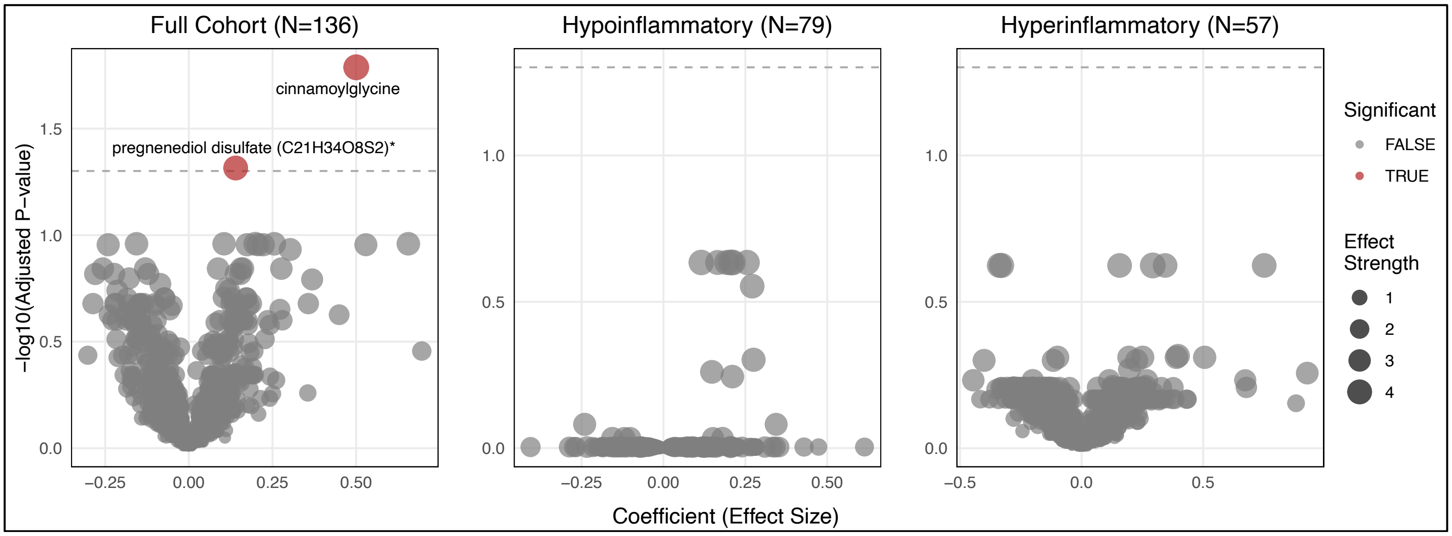

Supplemental Figure 6. Metabolite trajectories in 90-day non-survivors vs survivors. Analysis restricted to the subset who survived through Day 2 post-randomization. Plots display coefficients from linear mixed effects models testing the time-by-mortality interaction, controlling for randomization arm, age, sex, and BMI, and subject-level random effects. Points are sized by effect strength (|coefficient|/standard error), with significance determined by likelihood ratio tests against a model without the interaction term (FDR <0.05). Cinnamoylglycine and pregnenediol disulfate demonstrate the only significant divergent trajectories between survival groups, with stronger positive temporal trends in non-survivors.

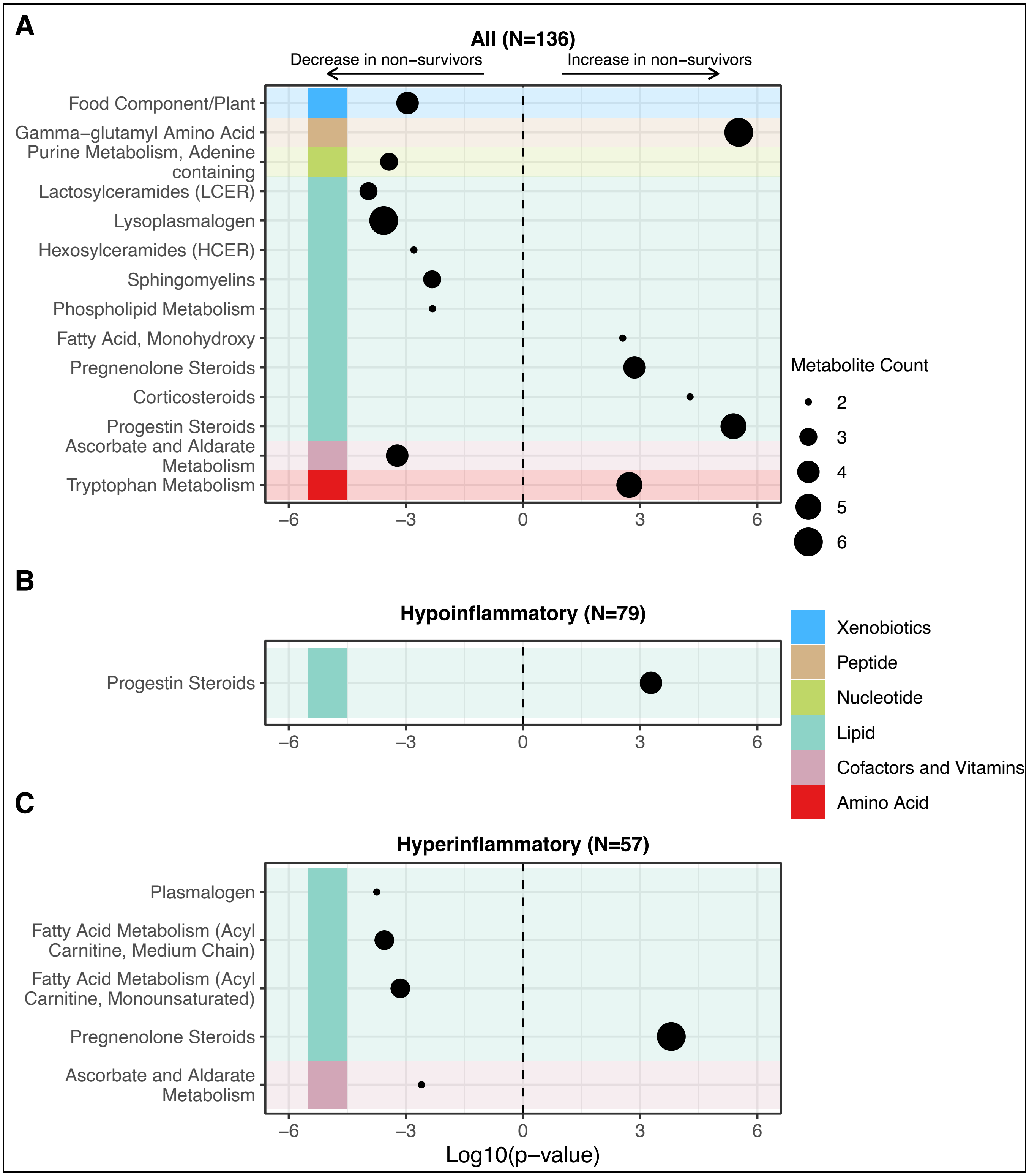

Supplemental Figure 7. Differential metabolite pathway enrichment in 90-day non-survivors versus survivors. Analysis restricted to the subset who survived through Day 2 post-randomization. Linear mixed effects models were used to determine differential rates of change over time controlling for randomization arm, age, sex, and BMI, and subject-level random effects. Model coefficients were converted to fold changes for enrichment analysis. Only metabolic classes with FDR adjusted p <0.05 are depicted. X-axis represents signed statistical significance (Log10[p-value]), with negative values indicating decreased abundance over time in non-survivors relative to survivors, and positive values indicating increased abundance. The size of each dot represents the number of metabolites contributing to each class.

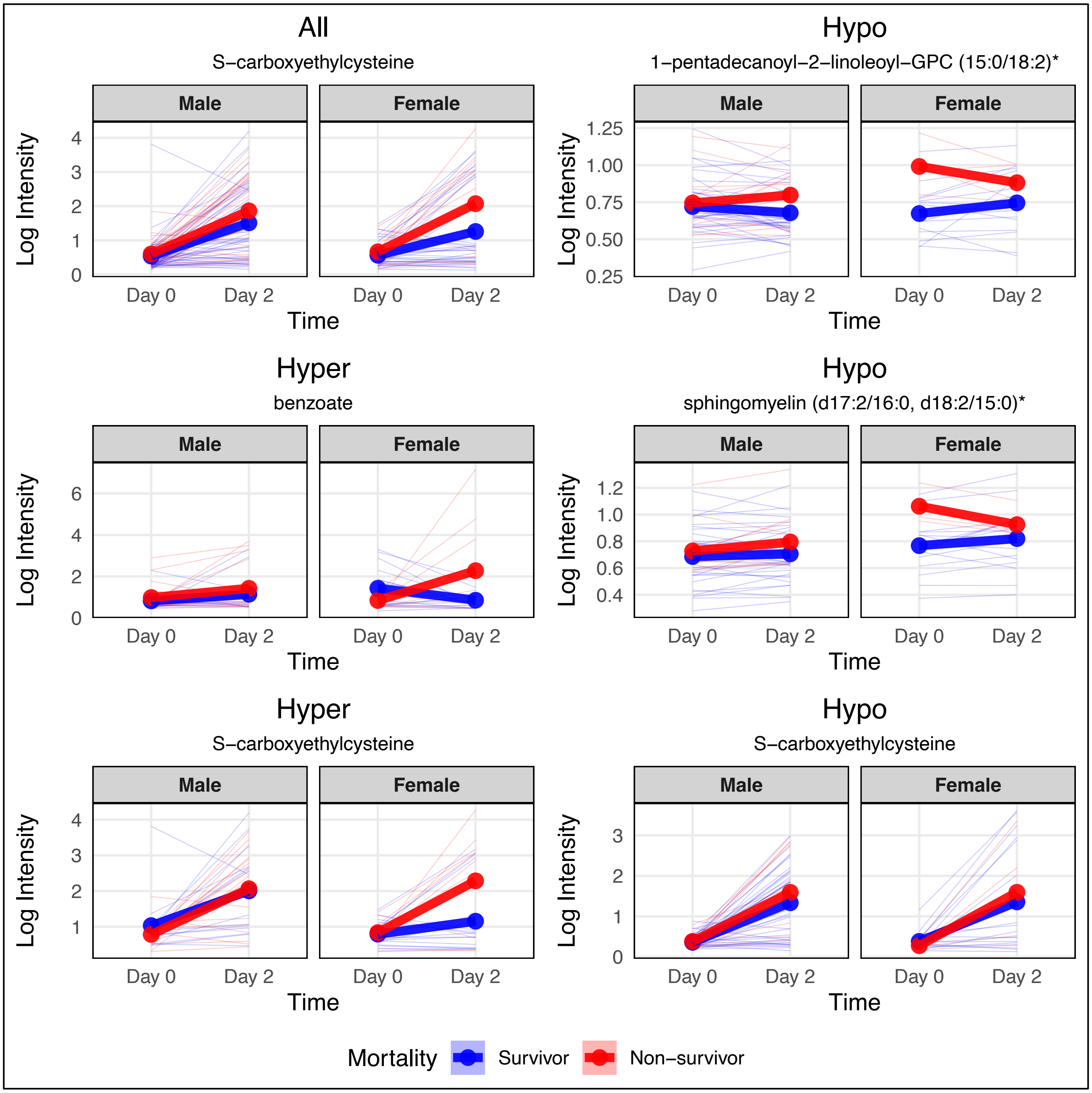

Supplemental Figure 8. Sex-specific differences in mortality-related metabolic trajectories. Metabolites showing significant three-way interactions between time, mortality, and sex. Mixed-effects models tested for differential metabolic trajectories by sex in relation to 90-day mortality. Log-transformed metabolite intensities are shown at Day 0 and Day 2 for survivors and non-survivors, stratified by sex. Only 6 of 982 metabolites demonstrated significant sex interactions (FDR <0.05).

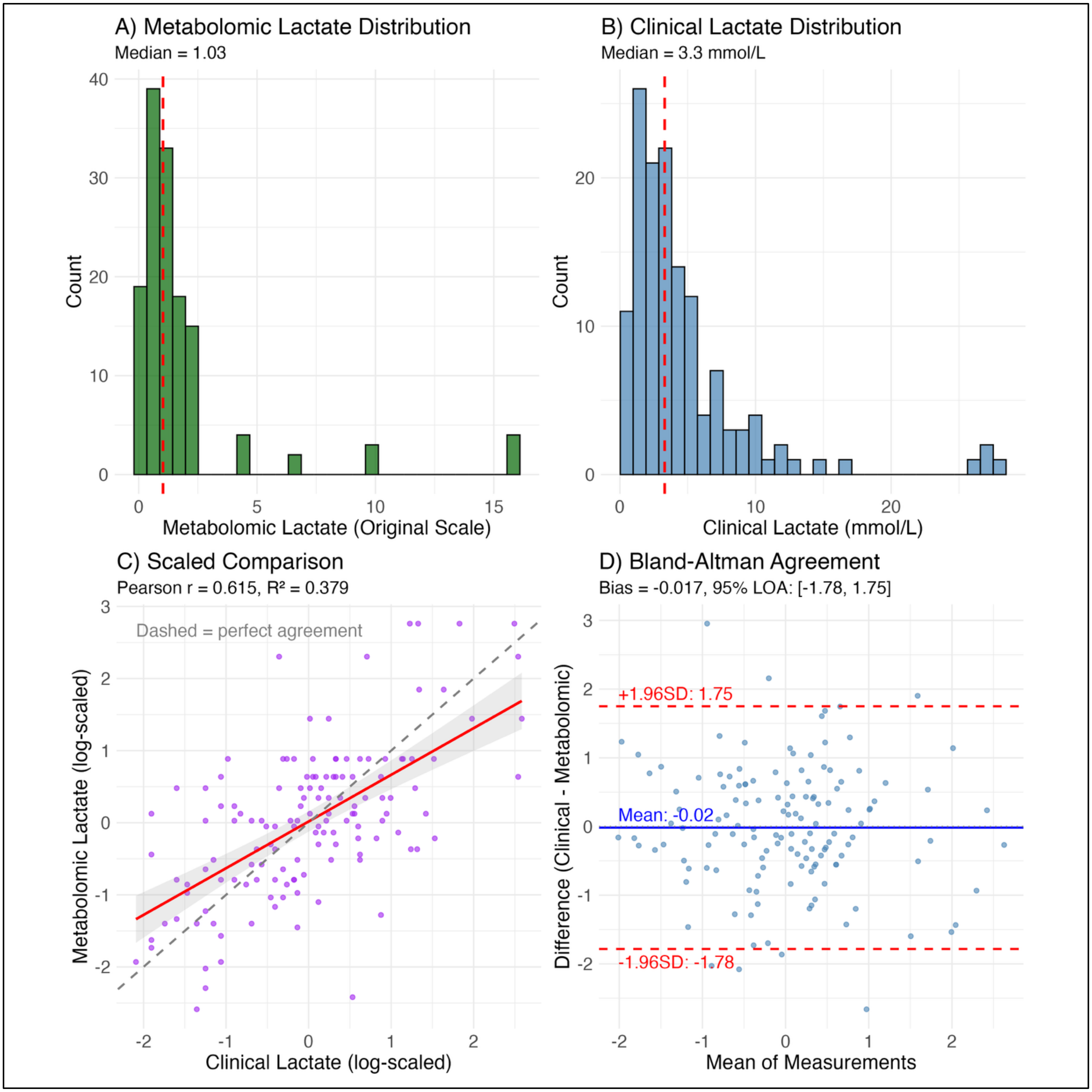

Supplemental Figure 9. Validation of metabolomic lactate against clinical measurements. Comparison of metabolomic and clinical lactate in EARLI patients with paired measurements available at baseline (n = 137). (**A**) Distributions at Day 1 for metabolomic lactate (batch-normalized area under the curve of peak intensity, median = 1.03). (**B**) Distributions at Day 1 for clinical lactate (mmol/L, median = 3.3). (**C**) Linear correlation after log-transformation and z-scaling (Pearson's r = 0.615, $R^2 = 0.379$, $p < 1\times10^{-4}$); dashed line represents perfect agreement. (**D**) Bland-Altman plot demonstrating agreement between scaled measurements (mean difference = -0.017, 95% LOA: [-1.78, 1.75]).

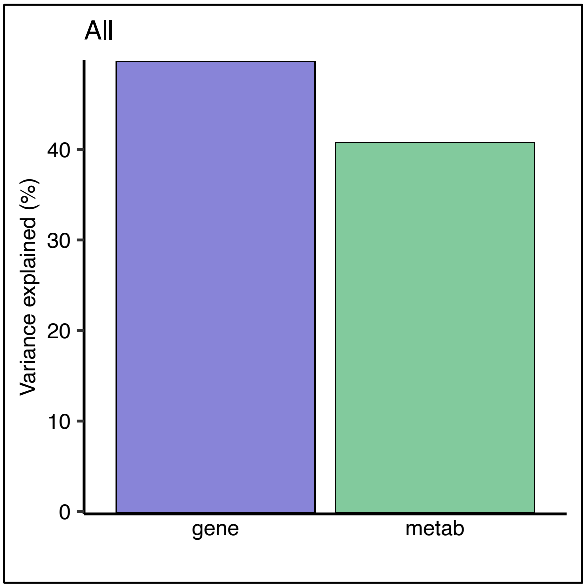

Supplemental Figure 10. **Variance explained per data modality in the ROSE MEFISTO model.** Plots depict proportion of total variance explained per data modality after applying MEFISTO to the full study cohort, n=160**.**

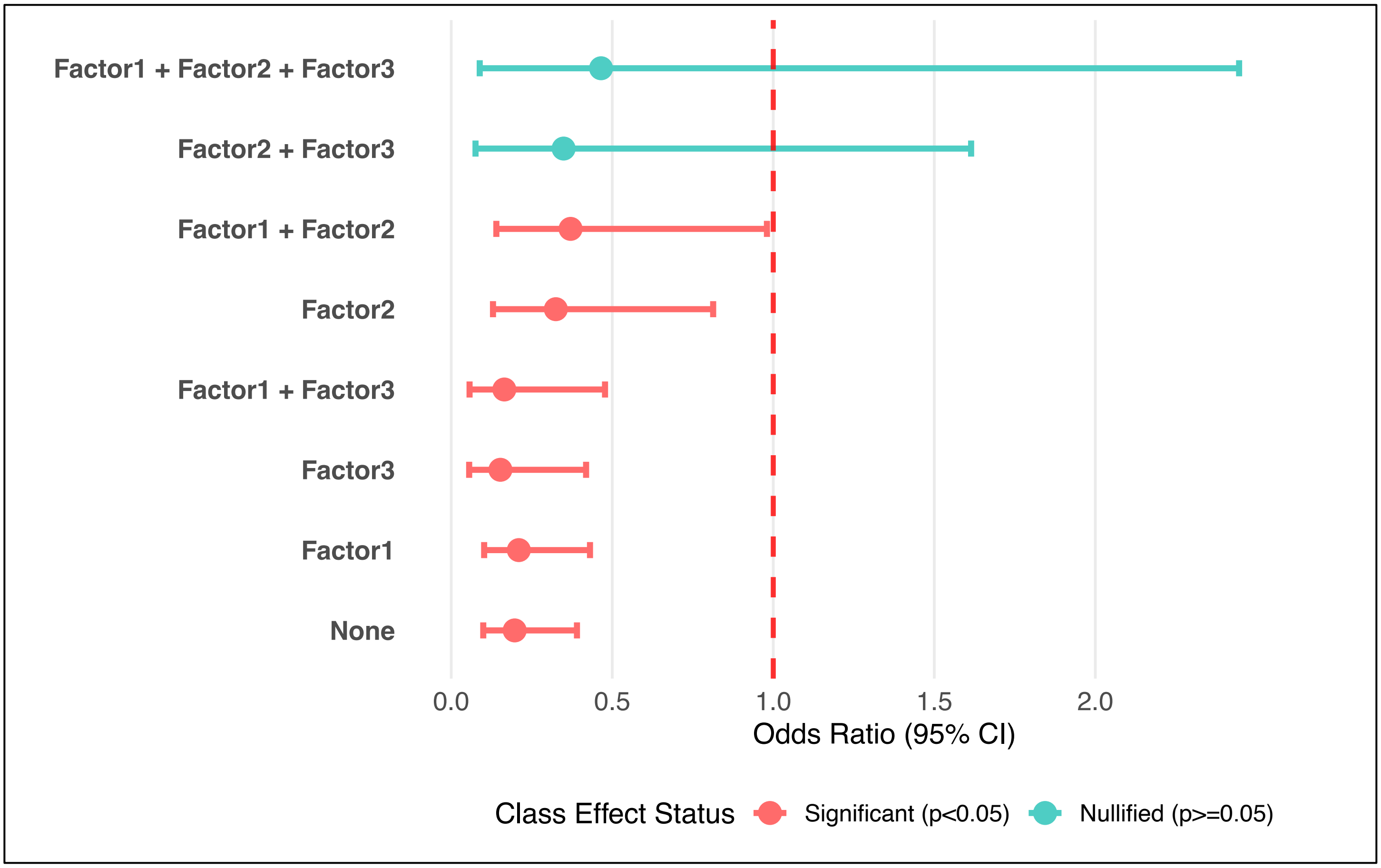

Supplemental Figure 11. Effect of MEFISTO factors on the association between LCA phenotype and mortality in the ROSE cohort (N = 160). Forest plot showing odds ratios (OR) and 95% confidence intervals (CI) for the association between hypoinflammatory ARDS and 90-day mortality after adjusting for different combinations of MEFISTO factors. "None" represents unadjusted model. Individual factors (Factor 1, Factor 2, Factor 3) and their combinations represent models where these factors are included as covariates.

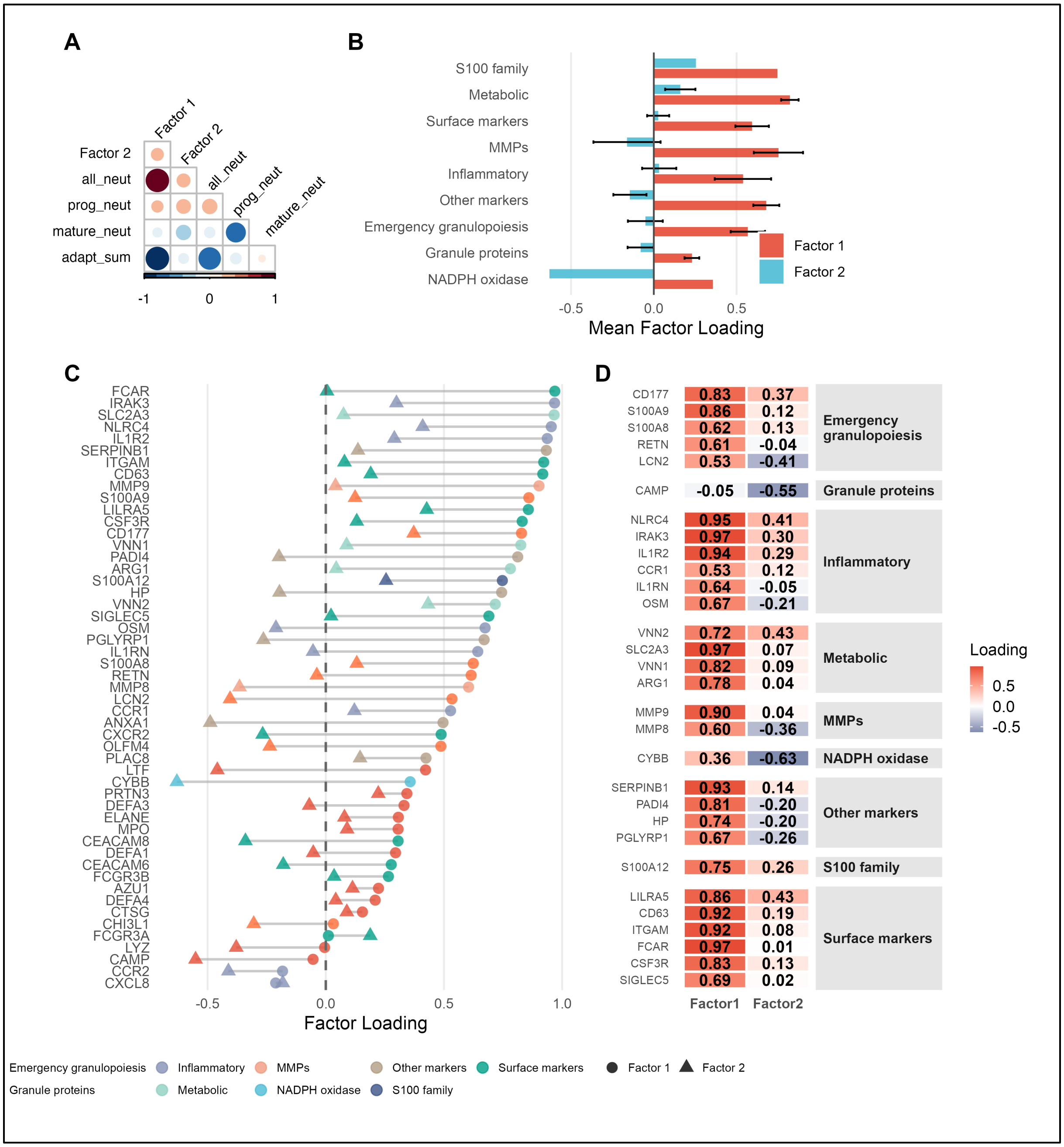

Supplemental Figure 12. Comparison of neutrophil-related gene expression in MEFISTO Factors 1 and 2. Genes were selected using the leading edge genes of the neutrophil degranulation Reactome pathway identified in gene set enrichment analysis of Factors 1 and 2, supplemented with top neutrophil-related genes from each factor. (**A**) Spearman correlation plot of Factors 1 and 2 with immune cell type proportions using CIBERSORTx. all_neut = all neutrophils, prog_neut = immature progenitor neutrophils, mature_neut = mature neutrophils, adapt_sum = adaptive immune cells. (**B**) Mean factor loadings by neutrophil gene functional category. Bars represent average absolute factor loading values across different neutrophil gene functional categories ± SEM. (**C**) Dumbbell plot comparing Factor 1 and Factor 2 loadings for individual neutrophil genes, arranged by Factor 1 loading strength. Genes are color-coded by functional category and shape-coded by factor. (**D**) Heatmap of the top 30 neutrophil genes ranked by maximum factor loading across both factors, grouped by functional category.

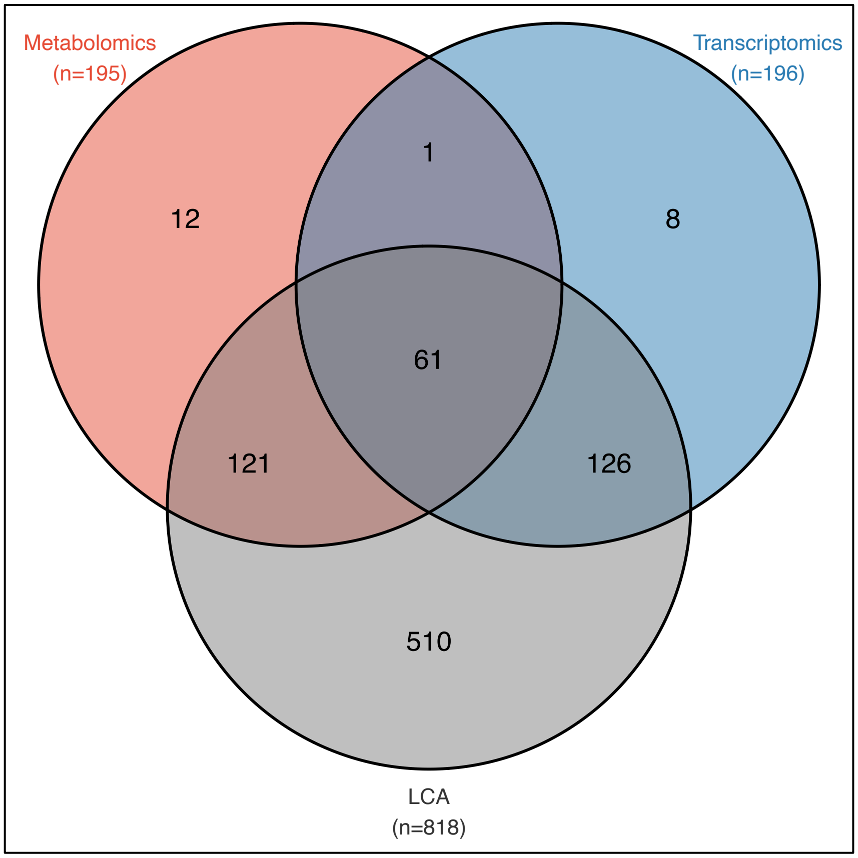

Supplemental Figure 13. EARLI validation cohort of patients with sepsis. Data collection included three overlapping groups: (1) transcriptomic data from study participants with hypotension or receiving invasive mechanical ventilation in the emergency room and sepsis (n=196); (2) protein biomarker data at enrollment available and used for latent class analysis (LCA; n=818); and (3) untargeted metabolic profiling from a randomly selected subset of sepsis patients with and without ARDS (n=195). Only patients with a diagnosis of sepsis within 2 days of enrollment in EARLI were included.

Supplemental Table 1: Characteristics of patients in the ROSE discovery cohort

	Hypoinflammatory (N=80)	Hyperinflammatory (N=80)	Total (N=160)	P-value[A]
**Age**, median (IQR)	58.5 (46 to 66)	58.5 (48 to 69)	58.5 (47 to 68)	0.38
**Sex**				0.14
Male	56 (70%)	46 (58%)	102 (64%)	
Female	24 (30%)	34 (42%)	58 (36%)	
**BMI**, median (IQR)	33 (27 to 38)	28 (23 to 35)	30 (25 to 37)	0.01
**Race**[B]				0.17
African American	10 (12%)	4 (5%)	14 (9%)	
White	61 (76%)	64 (80%)	125 (78%)	
Unknown	3 (4%)	1 (1%)	4 (2%)	
Missing	6 (7%)	11 (14%)	17 (11%)	
**Ethnicity**				0.72
Hispanic or Latino	6 (8%)	9 (11%)	15 (9%)	
Not Hispanic or Latino	69 (86%)	66 (83%)	135 (84%)	
Missing	5 (6%)	5 (6%)	10 (6%)	
**Randomization arm**				0.75
Neuromuscular blockade	42 (52%)	39 (49%)	81 (51%)	
Usual care	38 (47%)	41 (51%)	79 (49%)	
**APACHEIII**	84 $\pm$ 24	132 $\pm$ 31	110 $\pm$ 37	<0.0001
Missing[C]	30 (37%)	22 (27%)	52 (32%)	
**PaO2:FiO2[D],** median (IQR)				
Lowest in 24 hours	95 (76 to 126)	81 (67 to 105)	86 (71 to 115)	0.01
At enrollment	119 (92 to 140)	116 (93 to 138)	117 (92 to 140)	0.70
**GFR**, median (IQR)	78 (50 to 99)	26 (13 to 43)	48 (21 to 86)	<0.0001
**RRT at enrollment**	0	21 (26%)	21 (13%)	<0.0001
**Comorbidities**				
Acute kidney injury	12 (15%)	42 (52%)	54 (34%)	<0.0001
Hepatic failure with coma or encephalopathy	0	7 (9%)	7 (4%)	0.006
Cirrhosis	4 (5%)	15 (19%)	19 (12%)	0.007
Chronic dialysis	2 (2%)	5 (6%)	7 (4%)	0.28
Diabetes	20 (25%)	25 (31%)	45 (28%)	0.45
Hypertension	48 (60%)	42 (52%)	90 (56%)	0.47
Congestive heart failure	6 (7%)	5 (6%)	11 (7%)	1
Prior stroke	7 (9%)	5 (6%)	12 (7%)	0.78
Chronic pulmonary disease	26 (32%)	8 (10%)	34 (21%)	
Immunosuppression within the past 6 months	12 (15%)	12 (15%)	24 (15%)	1
Leukemia	0	6 (7%)	6 (4%)	0.01
AIDS	2 (2%)	2 (2%)	4 (2%)	1
**Primary cause of ARDS**				<0.0001
Pneumonia	49 (61%)	36 (45%)	85 (53%)	
Sepsis[E]	0	25 (31%)	25 (16%)	
Aspiration	20 (25%)	12 (15%)	32 (20%)	
Multiple transfusion	3 (4%)	2 (2%)	5 (3%)	
Trauma	5 (6%)	1 (1%)	6 (4%)	
Other	3 (4%)	4 (5%)	7 (4%)	

	Hypoinflammatory (N=80)	Hyperinflammatory (N=80)	Total (N=160)	P-value[A]
**Medications**				
Vasopressors	17 (21%)	69 (86%)	86 (54%)	<0.0001
Propofol	68 (85%)	48 (60%)	116 (72%)	0.002
Dexmedetomidine	14 (17%)	11 (14%)	25 (16%)	0.77
Corticosteroids[F]	19 (24%)	19 (24%)	38 (24%)	1
**Ventilator free days**, median (IQR)	20 (0 to 24)	0	0 (0 to 21)	<0.0001
**ICU free days,** median (IQR)	17.5 (0 to 23)	0 (0 to 8)	3.5 (0 to 19)	<0.0001
**Hospital free days**, median (IQR)	12 (0 to 18)	0 (0 to 0)	0 (0 to 15)	<0.0001
**28-day mortality**	19 (24%)	45 (56%)	64 (40%)	<0.0001
**90-day mortality**	19 (24%)	49 (61%)	68 (42%)	<0.0001

Numbers are presented as n (%) or mean ± SD unless otherwise stated. [A]As reported by the ROSE trial. [B]Determined via Welch's t-test for normally distributed continuous variables, Wilcoxon rank-sum for non-normally distributed continuous variables, and Chi-squared test or Fisher's exact test for categorical variables. [C]Data not collected or reported by the trial. [D]Lowest PaO2:FiO2 in the 24 hours preceding randomization, and PaO2:FiO2 on day of study enrollment. [E]If both pneumonia and sepsis were causes of lung injury, pneumonia was reported as primary. [F]Intravenous or enteral corticosteroids (≥ 20 mg methylprednisolone equivalents). BMI = body mass index, GFR = glomerular filtration rate, RRT = renal replacement therapy on day of enrollment

Supplemental Table 2. Proportion of differentially abundant metabolites between inflammatory phenotypes based on limma results at Day 0.

SUPER_PATHWAY	total_count[A]	significant_count[B]	proportion_significant
Energy	11	8	0.73
Carbohydrate	27	19	0.70
Nucleotide	40	24	0.60
Lipid	507	294	0.58
Amino Acid	210	119	0.57
Peptide	31	17	0.55
Partially Characterized Molecules	22	11	0.50
Cofactors and Vitamins	35	15	0.43
Xenobiotics	99	34	0.34

[A]Total count reflects the total number of metabolites within a given metabolic pathway entered into the analysis (after removal of metabolites with low variance across the cohort). [B]Significant count denotes the number of metabolites with FDR <0.05 as identified by limma adjusted for covariates age, sex, BMI, medications, comorbid liver disease, and GFR comparing Hyperinflammatory to Hypoinflammatory phenotypes

Supplemental Table 3. Proportion of differentially abundant metabolites between inflammatory phenotypes based on limma results at Day 2.

SUPER_PATHWAY	total_count[A]	significant_count[B]	proportion_significant
Energy	11	7	0.64
Partially Characterized Molecules	22	14	0.64
Carbohydrate	27	17	0.63
Lipid	507	291	0.57
Cofactors and Vitamins	35	19	0.54
Nucleotide	40	21	0.52
Amino Acid	210	83	0.39
Xenobiotics	99	35	0.35
Peptide	31	7	0.23

[A]Total count reflects the total number of metabolites within a given metabolic pathway entered into the analysis (after removal of metabolites with low variance across the cohort). [B]Significant count denotes the number of metabolites with FDR <0.05 as identified by limma adjusted for covariates age, sex, BMI, medications, comorbid liver disease, and GFR comparing Hyperinflammatory to Hypoinflammatory phenotypes

Supplemental Table 4. Proportion of differentially abundant metabolites between inflammatory phenotypes based on limma results at Day 0 in pneumonia only patients (N = 85)

SUPER_PATHWAY	total_count[A]	significant_count[B]	proportion_significant
Carbohydrate	27	14	0.52
Energy	11	5	0.45
Nucleotide	40	18	0.45
Amino Acid	210	81	0.39
Lipid	507	175	0.35
Peptide	31	9	0.29
Partially Characterized Molecules	22	6	0.27
Cofactors and Vitamins	35	7	0.20
Xenobiotics	99	12	0.12

[A]Total count reflects the total number of metabolites within a given metabolic pathway entered into the analysis (after removal of metabolites with low variance across the cohort). [B]Significant count denotes the number of metabolites with FDR <0.05 as identified by limma adjusted for covariates age, sex, BMI, medications, comorbid liver disease, GFR, and vasopressor use at study enrollment comparing Hyperinflammatory to Hypoinflammatory phenotypes

Supplemental Table 5. Proportion of differentially abundant metabolites between inflammatory phenotypes based on limma results at Day 2 in pneumonia only patients (N = 85)

SUPER_PATHWAY	total_count[A]	significant_count[B]	proportion_significant
Energy	11	7	0.64
Partially Characterized Molecules	22	14	0.64
Lipid	507	244	0.48
Nucleotide	40	17	0.42
Carbohydrate	27	9	0.33
Cofactors and Vitamins	35	11	0.31
Amino Acid	210	60	0.29
Xenobiotics	99	23	0.23
Peptide	31	3	0.10

[A]Total count reflects the total number of metabolites within a given metabolic pathway entered into the analysis (after removal of metabolites with low variance across the cohort). [B]Significant count denotes the number of metabolites with FDR <0.05 as identified by limma adjusted for covariates age, sex, BMI, medications, comorbid liver disease, GFR, randomization arm and vasopressor use at study enrollment comparing Hyperinflammatory to Hypoinflammatory phenotypes

Supplemental Table 6. Proportion of differentially abundant metabolites between inflammatory phenotypes based on limma results at Day 0 adjusting for renal replacement therapy at enrollment

SUPER_PATHWAY	total_count[A]	significant_count[B]	proportion_significant
Carbohydrate	27	18	0.67
Energy	11	7	0.64
Nucleotide	40	23	0.57
Lipid	507	286	0.56
Peptide	31	17	0.55
Amino Acid	210	111	0.53
Partially Characterized Molecules	22	10	0.45
Cofactors and Vitamins	35	14	0.40
Xenobiotics	99	30	0.30

[A]Total count reflects the total number of metabolites within a given metabolic pathway entered into the analysis (after removal of metabolites with low variance across the cohort). [B]Significant count denotes the number of metabolites with FDR <0.05 as identified by limma adjusted for covariates age, sex, BMI, medications, comorbid liver disease, GFR, randomization arm and renal replacement therapy at study enrollment (binary variable) comparing Hyperinflammatory to Hypoinflammatory phenotypes

Supplemental Table 7. Proportion of differentially abundant metabolites between inflammatory phenotypes based on limma results at Day 2 adjusting for renal replacement therapy at enrollment

SUPER_PATHWAY	total_count[A]	significant_count[B]	proportion_significant
Partially Characterized Molecules	22	14	0.64
Lipid	507	264	0.52
Nucleotide	40	20	0.50
Energy	11	5	0.45
Carbohydrate	27	11	0.41
Cofactors and Vitamins	35	13	0.37
Amino Acid	210	70	0.33
Xenobiotics	99	29	0.29
Peptide	31	3	0.10

[A]Total count reflects the total number of metabolites within a given metabolic pathway entered into the analysis (after removal of metabolites with low variance across the cohort). [B]Significant count denotes the number of metabolites with FDR <0.05 as identified by limma adjusted for covariates age, sex, BMI, medications, comorbid liver disease, GFR, randomization arm and renal replacement therapy at study enrollment (binary variable) comparing Hyperinflammatory to Hypoinflammatory phenotypes

Supplemental Table 8. MEFISTO model variance explained for each data type and by each factor.

Factor	Gene_Variance	Gene_Percent_of_Total	Metab_Variance	Metab_Percent_of_Total
Factor1	17.37	35.01	4.10	10.08
Factor2	0.80	1.62	17.65	43.41
Factor3	10.89	21.94	6.11	15.02
Factor4	0.27	0.54	9.27	22.80
Factor5	6.58	13.26	0.62	1.53
Factor6	3.77	7.59	1.85	4.55
Factor7	3.52	7.10	1.76	4.33
Factor8	2.75	5.55	0.89	2.19
Factor9	2.08	4.20	1.25	3.08
Factor10	1.74	3.52	1.22	3.01

Supplemental Table 9. Characteristics of participants with biological data in the EARLI validation cohort

	Transcriptomic Cohort (N=196)	Metabolomic Cohort (N=195)	Overlapping Cohort (N=62)
**Age**	65 ± 15	67 ± 14	69 ± 11
**Race[A]**			
White	75 (38%)	104 (53%)	27 (43%)
African American	34 (17%)	24 (12%)	11 (18%)
Asian	60 (31%)	55 (28%)	20 (32%)
American Indian	1 (0.5%)	0	0
Unknown	26 (13%)	12 (6%)	4 (6%)
**Sex**			
Male	118 (60%)	107 (55%)	37 (60%)
Female	78 (40%)	88 (45%)	25 (40%)
**BMI**	24 (21 to 29)	24 (20 to 28)	24 (21 to 28)
**Patient Category**			
Medicine	175 (89%)	159 (81%)	53 (85%)
General Surgery	3 (1%)	7 (4%)	2 (3%)
Cardiothoracic Surgery	1 (0.5%)	1 (0.5%)	1 (2%)
Vascular Surgery	1 (0.5%)	0	0
Transplant Surgery	0	1 (0.5%)	0
Neurology	0	1 (0.5%)	0
Cardiology	8 (4%)	8 (4%)	2 (3%)
Other	8 (4%)	18 (9%)	4 (6%)
**Comorbidities**			
Congestive heart failure	30 (15%)	29 (15%)	9 (14%)
Cardiovascular disease	18 (9%)	21 (11%)	6 (10%)
Hypertension	88 (45%)	127 (65%)	36 (58%)
Chronic lung disease	63 (32%)	61 (31%)	22 (35%)
Chronic liver failure	5 (3%)	3 (1%)	2 (3%)
Cirrhosis	20 (10%)	12 (6%)	5 (8%)
Diabetes	44 (22%)	55 (28%)	1 (26%)
Malignancy	35 (18%)	65 (33%)	15 (24%)
HIV/AIDS	13 (7%)	14 (7%)	8 (13%)
Solid organ transplant	5 (3%)	9 (5%)	3 (5%)
Chronic immunosuppression	12 (6%)	23 (12%)	5 (8%)
Other immunocompromised	5 (3%)	18 (9%)	1 (2%)
ESRD	11 (6%)	12 (6%)	5 (8%)
Dialysis	14 (7%)	9 (5%)	4 (6%)
CKD	21 (11%)	36 (18%)	8 (13%)
**Sepsis type**			

	Transcriptomic Cohort (N=196)	Metabolomic Cohort (N=195)	Overlapping Cohort (N=62)
Pulmonary and non-pulmonary	12 (6%)	18 (9%)	3 (5%)
Non-pulmonary	70 (36%)	53 (27%)	16 (26%)
Pulmonary	108 (55%)	113 (58%)	41 (66%)
Unclear source	6 (3%)	11 (6%)	2 (3%)
**ARDS, Berlin**	82 (42%)	88 (45%)	37 (60%)
**ARDS Severity**[B]			
Mild	16 (8%)	6 (3%)	3 (5%)
Moderate	39 (20%)	47 (24%)	21 (34%)
Severe	27 (14%)	35 (18%)	13 (21%)
**Acute kidney injury**	98 (50%)	108 (55%)	39 (63%)
**GFR**	56 (26 to 89)	50 (26 to 81)	56 (31 to 76)
**On vasopressors**	81 (41%)	52 (27%)	24 (39%)
**APACHE III**	115 $\pm$ 38	100 $\pm$ 40	121 $\pm$ 35
**SAPS II**	63 $\pm$ 20	56 $\pm$ 22	67 $\pm$ 19
**LCA Phenotype Assigned**	187 (95%)	182 (93%)	61 (98%)
Hyperinflammatory	76 (39%)	61 (31%)	27 (43%)
Hypoinflammatory	111 (57%)	121 (62%)	34 (55%)
**Outcome**			
Died	71 (36%)	72 (37%)	31 (50%)
Survived	125 (64%)	123 (63%)	31 (50%)

Numbers are presented as n (%) for categorical variables and mean $\pm$ SD for continuous variables. [A]As designated in the medical record. [B]Severity defined using the PaO2:FiO2 or the SpO2:FiO2 if the former was missing. BMI = body mass index, CKD = chronic kidney disease, ESRD = end-stage renal disease, GFR = glomerular filtration rate.
